# Contribution of genetic ancestry and polygenic risk score in meeting vitamin B12 needs in healthy Brazilian children and adolescents

**DOI:** 10.1038/s41598-021-91530-7

**Published:** 2021-06-07

**Authors:** Carlos Alessandro Fuzo, Fábio da Veiga Ued, Sofia Moco, Ornella Cominetti, Sylviane Métairon, Solenn Pruvost, Aline Charpagne, Jerome Carayol, Raul Torrieri, Wilson Araujo Silva, Patrick Descombes, Jim Kaput, Jacqueline Pontes Monteiro

**Affiliations:** 1 Department of Clinical Analyses, Toxicology and Food Sciences, School of Pharmaceutics Sciences, University of São Paulo, Ribeirão Preto, Brazil; 2 Department of Pediatrics and Department of Health Sciences, Ribeirão Preto Medical School, Nutrition and Metabolism Section, University of São Paulo, Avenida Bandeirantes, 3900, Bairro Monte Alegre, Ribeirão Preto, SP 14040-900 Brazil; 3 Department of Chemistry and Pharmaceutical Sciences, Amsterdam Institute for Molecular and Life Sciences, Vrije Universiteite Amsterdam, Amsterdam, The Netherlands; 4 Nestlé Research, Société Des Produits Nestlé SA, EPFL Innovation Park, H, 1015 Lausanne, Switzerland; 5 Center for Medical Genomics, Ribeirão Preto Medical School Hospital, University of São Paulo, Ribeirão Preto, Brazil; 6 Department of Genetics, Ribeirão Preto Medical School, University of São Paulo, Ribeirão Preto, Brazil; 7 Vydiant, Folsom, CA USA; 8 Present Address: Sophia Genetics, Campus Biotech, 1202 Geneva, Switzerland

**Keywords:** Computational biology and bioinformatics, Genetics, Systems biology, Medical research, Molecular medicine, Risk factors

## Abstract

Polymorphisms in genes related to the metabolism of vitamin B12 haven’t been examined in a Brazilian population. To (a) determine the correlation between the local genetic ancestry components and vitamin B12 levels using ninety B12-related genes; (b) determine associations between these genes and their SNPs with vitamin B12 levels; (c) determine a polygenic risk score (PRS) using significant variants. This cross-sectional study included 168 children and adolescents, aged 9–13 years old. Total cobalamin was measured in plasma. Genotyping arrays and whole exome data were combined to yield ~ 7000 SNPs in 90 genes related to vitamin B12. The Efficient Local Ancestry Inference was used to estimate local ancestry for African (AFR), Native American, and European (EUR). The association between the genotypes and vitamin B12 levels were determined with generalized estimating equation. Vitamin B12 levels were driven by positive (EUR) and negative (AFR, AMR) correlations with genetic ancestry. A set of 36 variants were used to create a PRS that explained 42% of vitamin level variation. Vitamin B12 levels are influenced by genetic ancestry and a PRS explained almost 50% of the variation in plasma cobalamin in Brazilian children and adolescents.

## Background

Public health recommendations for the intake of micronutrients are designed to meet the requirements of the majority (97–98%) of healthy individuals within a population group^1^. However, no standard approach has been developed for deriving vitamin and mineral recommendations^2,3^, and large variations exist across countries causing confusion among consumers, food producers, and policy makers. Advising diets and food patterns for individuals of different ages, conditions (e.g., pregnancy, lactation, athletes), and diseases is even more challenging because physiologies are affected by interactions between genetic makeup and environmental conditions which also change over time.

A large number of studies have focused on how genetic variation might affect micronutrient metabolism, clinical and metabolomic measurements, and phenotypic expression^4–6^ with the goal of personalizing recommendations based on genetic variation^7^. Over 2300 publications on associations between single-nucleotide polymorphisms (SNPs) in candidate genes involved in nutrient (including vitamins and minerals) metabolism or response, as well as with disease^8,9^ have been published since 2001^10^. The majority of reports found statistical correlations but effect sizes were uniformly very small (usually < 1% of total phenotype) and reproducibility between studies is low. These results can be explained by gene–gene interactions, especially between different ancestral populations, and variation in diet and environmental/lifestyle factors that underlay gene-nutrient interactions^11–13^. In addition, reductionistic approaches ignore the many metabolite–protein (and therefore gene) and protein–protein interactions (i.e., gene–gene interactions) that produce an observable and measurable phenotype^14^.

More extensive omics measurements^4,15,16^ and systems biology approaches^11^ may provide a pathway to map the interactions between genotype, environmental factors, nutritional intakes and more exhaustive physiological analyses in different ethnic populations^4,12,17–20^. We tested the influence of genetic ancestry on baseline vitamin levels and found negative association between Native American (AMR) ancestry and vitamin B12 levels^17^. Genetic ancestry is an important cofounding variable for each specific phenotype, as admixed individuals inherit different combinations of causative variants based on the ancestral population in which those variants were present^21^.

Vitamin B12 is an essential water-soluble B vitamin that plays an elemental role in DNA synthesis, methylation reactions, and genomic stability, as well as mitochondrial metabolism^22–25^. Vitamin B12 may impact cognitive function^12,26^, cardiovascular disease through its role in homocysteine metabolism, and the phosphatidylethanolamine to phosphatidylcholine pathway, thereby influencing the concentrations of polyunsaturated fatty acids in plasma and in red blood cells^27,28^.

Inadequate intake or bioavailability and malabsorption are causes of vitamin B12 deficiency. Sub-clinical deficiency may affect 2.5–26% of the population, depending on the definition, as the cut-offs are undefined^29^. Studies with children in Brazil have shown different vitamin B12 deficiency rates, such as 3.7%^30^ 11.7%^31^ and 15%^32^. The mandatory flour fortification with folic acid in Brazil has proved to be effective in increasing serum folate concentrations in children and adolescents^33^ but some researchers speculate that excess folate may interfere with vitamin B12 metabolism and may worsen the functional consequences of impaired vitamin B12 deficiency^34^. Although some Brazilian authors suggest that vitamin B12 deficiency is practically nonexistent in children^35^, the screening for vitamin B12 deficiency may be particularly relevant in our population because the combination of high serum folate and normal vitamin B12 status has been associated with a lower frequency of anemia in these children^30,32^. Severe deficiency without treatment may lead to high homocysteine levels in Brazilian children^35^.

Enzymes and transporter proteins play important roles in metabolism of vitamin B12 and, therefore in its status. Polymorphisms in genes related to the metabolism of vitamin B12 have been examined in several studies, including genome-wide association ones^36–43^, but the results are still ambiguous or inclusive and have not been well defined in Brazilian population. Determining the genetic factors that may influence vitamin B12 requirements may help form the basis for personalizing B12 intake recommendations. Variants within B12-related genes that are significantly associated with vitamin B12 levels^36,37^ may be used, in a middle out approach^44^, to construct a polygenic risk score (PRS) after adjusting for cofounding variables, such as genetic ancestry. The PRS of an individual is defined as a quantitative measure of the total genetic risk burden of the phenotype over multiple susceptibility loci^45^. Genetic risk estimation is the most basic measurable contributor to common heritable disease risk. Recent studies suggest that, for a subset of diseases, polygenic risk profiling provides personal and clinical utility as well as for therapeutic intervention and/or life planning^46^.

Genetic makeup may play an important role in which populations or subgroups will be more sensitive to differences in B12 vitamin availability, and also explain the prevalence of hyperhomocysteinemia and methylmalonic acidemia since most Western diets provide adequate supplies of B12, B6 and folic acid^12^. The present study aimed to (i) determine the correlation between the local ancestry components (AFR, AMR, EUR) and baseline vitamin B12 levels using SNPs in ninety B12-related genes; (ii) determine associations of 90 vitamin B12 related genes and vitamin B12 levels using generalized estimation equation, adjusted for sex, BMI, age, healthy eating index (HEI) and mean ancestry; (iii) determine a PRS using only the variants within the 90 related vitamin B12 genes that were significantly associated with vitamin B12 levels.

## Methods

### Study population and design

The data described in this cross-sectional study were from the crossover N-of-1 micronutrient intervention study previously reported^17^. Briefly, a six-week multivitamin/mineral intervention was conducted in 9–13-year-old children and adolescents. Participants were: (i) their own control (N-of-1); (ii) monitored for compliance; and (iii) measured for food intake, anthropometric and metabolites in plasma and RBCs, at baseline (Visit 1), post intervention (Visit 2), and following a 6-week washout (Visit 3) in two consecutive years, 2013 and 2014. Genetic profile was analyzed at baseline. To avoid the influence of these supplements on plasma B12 vitamin levels, only the baseline data (Visit 1) were used in the analyses described here.

Data collection was performed at the Ribeirão Preto Medical School Hospital (HCFMRP-USP), University of São Paulo, Brazil. The study was approved by the internal ethics committee (Process HCRP No. 14255/2010) and by the National Research Ethics Commission (No. 00969412.6 CAAE. 0000.5440). The trial was registered on ClinicalTrials.gov (NCT01823744–April 4th 2013). All the experiments were performed in accordance with relevant guidelines and regulations. The participants were informed about the purpose and procedures of the study and signed a statement of informed consent. Parents of each participant signed informed consent^17,28^.

Participants in this study were clinically stable children and adolescents, i.e., without injury, or infectious diseases, as specified in exclusion criteria. Children and adolescents aged 9–13 years, were recruited from three schools in the west side of Ribeirão Preto. This municipality is in the northeastern region of the state of São Paulo in Brazil^17,28^. Exclusion criteria were individuals: (i) with one or more episodes of axillary temperature higher than 37 °C in the 15 days preceding the blood collection; (ii) with three or more episodes of liquid stools in the 24 h before assessment; (iii) with intake of any kind of vitamin or mineral supplement; (iv) on a supervised diet for reducing weight or any other type of dietary restriction; (v) with a diagnosis of chronic disease that may interfere with data collection; and (vi) who participated in another clinical trial in the four weeks preceding the study.

The upper age cut-off was 13 years, 11 months and 29 days at registration visit (Visit 1). Individuals in all weight groups were included. A total of 280 participants met the inclusion criteria. After removing siblings and outliers of clinical and vitamins levels, 168 participants were considered for analysis in the present study^17,28^.

### Anthropometry and socioeconomic rating

A dietitian measured height and weight of participants immediately after fasted blood collection (12 h) and BMI was calculated according to World Health Organization (WHO)^47^. The questionnaire for socioeconomic rating was from *Associação Brasileira de Empresas de Pesquisa*^48^.

### Determination of metabolites levels

The analysis of metabolites investigated in this study has already been detailed described by Mathias et al.^17^ and by Ued et al.^28^. Total cobalamin and folate in plasma using AM-396 and MonoBind ELISA (Folate/Vitamin B12 Anemia Panel VAST test system, Monobind, Lake Forest, CA 92630, USA) was measured by Vitas (Norway). Vitamin B12 levels were defined as follow^49^: Low plasma levels (< 148 pmol/L), normal range (≥ 148 < 295 pmol/L); above normal range ≥ 295 pmol/L). Total homocysteine (tHcy) was measured in red blood cells (RBC) by liquid chromatography tandem mass spectrometry (LC–MS/MS) according to da Silva et al. 2016^50^. Riboflavin and pyridoxal were analyzed in plasma by LC–MS/MS according to Meisser et al.^51^.

### Genotype and exome sequencing data

Genotype data were generated using the HumanOmni5Exome 4v1_A (IlluminaTM) according to the Infinitum^®^ LCG Quad Assay Protocol Guide^52^. Exome capture and sequencing used 3 ug of genomic DNA extracted from blood which was fragmented on a E220 Covaris to an average size of 150–200 bp. Following purification of the fragmented DNA using Ampure XP magnetic beads (Agencourt), libraries were prepared using the Agilent library kit (SureSelectXT Automated Target Enrichment for Illumina Paired-End Multiplexed Sequencing Automated using Agilent NGS Workstation Option B, Version F.3, October 2014). Agilent SureSelect V5 plus UTR kit, which targets 75 Mb of genomic regions, was used for exome capture following manufacturer’s instructions. Pools of 96 libraries were assembled and pooling homogeneity was evaluated by sequencing on MiSeq Nano Kit V2 with around 1.2 M reads pass filter. Deep sequencing was next performed on HiSeq2500 (Illumina) with PE 125 reads using V3 chemistry, with targeted mean exon coverage of 100× with a minimum threshold set at 70×. The SNPs were assigned to the coordinates of Genome Reference Consortium Human genome build 37 (GRCh37)^53^.

### Local ancestry determination and their correlation with vitamin B12 levels

The Efficient Local Ancestry Inference (ELAI)^54^ was used to estimate local ancestry for each individual for three-way admixture for the following ancestral groups (components): African, Native American, and European. A total of 76,307 SNPs from samples of this study population mapped to both Human Genome Diversity Project (HGDP)^55^ and 1000 Genomes Project^56^ reference populations, according to Mathias et al.^17^. Averages for ancestry were calculated for each SNP from each individual, with 10 calculations at each of 20 steps along each chromosome by specifying the number of admixing generations equal to 15 spanning from a defined upper-layer cluster equal to 3 and a lower-layer cluster equal to 15^54^. In order to check for consistency, the ADMIXTURE tool^57^ was also used to determine the global ancestry to compare with mean ancestry values obtained from local ancestry. Spearman correlation was determined between the local ancestry components (AFR, AMR, EUR) and baseline vitamin B12 levels with the *p* value calculated after 10,000 permutations (*p*_perm_). The results were used to generate a *correlation score* defined as – log_10_(*p*_perm_) multiplied by the sign of the correlation. Significance was accepted at *p*_perm_ < 0.1.

### Statistical analysis on genes related to vitamin B12 levels

A list of genes related to vitamin B12 levels was obtained from a gene set called *vitamin B12 deficiency* from the DISEASES resource^36^, and includes 76 genes from text mining and manually curated disease-gene associations. This list was expanded with 14 other B12-related genes described in a recent review^37^ resulting in a total of 90 B12-related genes (Additional file 2: Table S1 and S2). The DISEASES resource^36^ is a comprehensive freely available database of disease-gene associations. The associations are extracted through automatic text mining with evidence from databases with permissive licenses, namely manually curated associations from Genetics Home Reference (GHR) and UniProt Knowledgebase (UniProtKB), GWAS results from DistiLD, and mutation data from Catalog of Somatic Mutations in Cancer (COSMIC). A recent review of 16 studies of vitamin B12-related genes provided other SNPs with statistically significant associations with blood vitamin B12 concentrations^37^. The populations included in the studies were African American, Brazilian, Canadian, Chinese, Danish, English, European, Icelandic, Indian, Italian, Latino, Northern Irish, Portuguese and residents in the United States.

The coordinates of the loci from transcription start to end sites were obtained from NCBI (37.3) gene definitions^58^ and were employed to mapping the variants observed in the samples. Variants were between 5 kb upstream from the start coordinate to 1.5 kb past the end coordinate were annotated to the respective genes. Variants extracted from exome data with VCF Tools^59^ were merged with genotype SNPs identified with PLINK^60^. Genetic ancestry per gene was determined as the mean local ancestry values calculated at SNP positions within each gene region. When no SNP determined local ancestry was within the region of the gene, the gene borders were interactively increased by 1 kb in order to search for average ancestry in its neighborhood (Additional file 2: Table S1). Spearman correlation was determined between the local ancestry components (AFR, AMR, EUR) per each 90 B12-related gene and baseline vitamin B12 levels with the *p*_perm_ value calculated after 10,000 permutations. The genes were correlated with vitamin B12 levels within a significant level of *p*_perm_ < 0.1.

The levels of vitamin B12 were associated with variants mapped to previously defined gene regions. The software PLINK 1.9^60^ was used for quality control (QC) analysis excluding variants with Hardy–Weinberg equilibrium with exact test *p*-values < 10^–^^3^ and with minor allele frequency (MAF) < 0.01. The association between the genotypes and vitamin B12 levels were determined with generalized estimating equation (GEE) employing the function geese of *geepach* R library^61^ and using as covariates sex, BMI, age, total HEI and mean ancestry components (AFR, AMR, and EUR) per each individual. Vitamin B12 levels were log_2_ transformed in order to obtain a normal distribution. The SNPs in the 90 B12 related genes were then clumped into blocks of linkage disequilibrium (LD) based on an r^2^ threshold > 0.2 within a window of 250 kb. For each clumped block the more significant associated SNP was denoted as SNP_ref_. The *p*-values for the resulting list of SNP_ref_ were corrected (*p*_adj_) for multiple testing using the Benjamini and Hochberg method^62^ and a significant association was accepted at *p*_adj_ < 0.1.

String v.11 was used to interrogate known protein–protein network associations^63^ and p-value was adjusted for multiple comparisons (False Discovery Rate—FDR). A significant association was accepted at *p* < 0.05.

### Polygenic risk score (PRS) construction

SNPs within B12-related genes that were significantly associated with vitaminB12 levels in GEE analysis were used to construct a PRS. The PRS was computed by summing the number of risk alleles (0, 1, or 2) weighted by the effect size from GEE results^64^.

### Stratification of individuals into PRS terciles

The individuals were stratified into terciles based on the PRS in order to determine how variables related to individuals, such as age, sex, body mass index, total HEI, meat and milk intake (HEI components), socioeconomic status and levels of vitamin B12, tHcy, folate, pyridoxal and riboflavin, are distributed across the terciles. Kruskal–Wallis was employed to evaluate the significance of differences among terciles within a significance level of 0.05.

### Ethics approval and consent to participate

The study was approved by the internal ethics committee (Process HCRP No. 14255/2010) and by the National Research Ethics Commission (No. 00969412.6CAAE.0000.5440). The trial was registered on ClinicalTrials.gov (NCT01823744). The participants were informed about the purpose and procedures of the study and signed a statement of informed consent. Parents of each participant signed informed consent.

## Results

### Ancestry along the genome and their per gene correlation with phenotype

After removing siblings and outliers of clinical and vitamins levels, 168 participants were considered for analysis in the present study. The investigation of local ancestry along the genome was motivated by previous results^17^ that showed a negative association between baseline levels of vitamin B12 with AMR. First, the mean values for the local ancestry components AFR, AMR and EUR calculated for each individual were compared with global ancestry from ADMIXTURE tool to check for consistency of the ancestry levels obtained in Mathias et al.^17^. This comparison shows that both tools generated the same patterns of ancestry contributions (Additional file 1: Figure S1).

The highest ancestral contribution throughout the autosomal chromosomes is from EUR with mean values ranging from 0.54 to 0.67, followed by AFR (0.18–0.31), and AMR (0.10–0.21) (Additional file 1: Figure S2), consistent with the overall genome-wide mean ancestry for each component.

**Figure 1:**
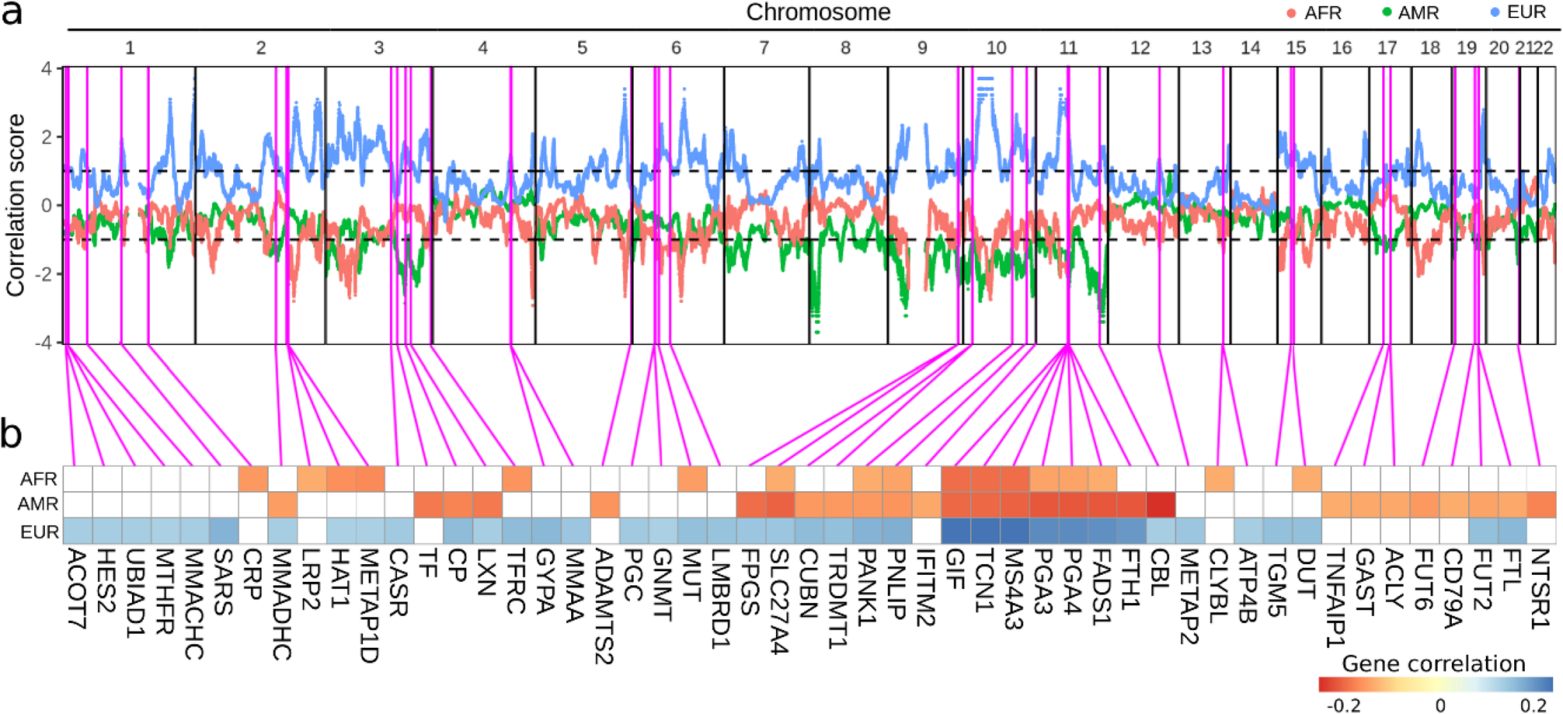
Ancestry results of three-way admixture AFR, AMR, and EUR. Mean local ancestry for individuals alongside autosomal chromosomes (**a**). Results of 10,000 permutations per each correlation between three-way local ancestry for (**b**) genes in Table 1 and vitamin B12 baseline levels. The position of the gene is designated by the vertical line (magenta). White cells indicate no significant correlation at *p*_perm_ < 0.1.

The decomposition of ancestry components along the genome was used to identify regions associated with variations in the levels of vitamin B12. For this purpose, the local ancestry along the chromosomes for all children were correlated with vitamin B12 resulting in *correlation score*s as defined in methods (Fig. 1a). The results showed that baseline vitamin B12 levels were driven by positive correlations with EUR and negatively correlated with AFR and AMR ancestries. These associations corroborated the observed negative association with AMR in Mathias et al.^17^ study. These results also revealed that genetic local ancestry has an important role in vitamin B12 levels.

We also determined the mean ancestry for regions encoding each of the 90 genes involved in B12 metabolism and the genes that carried ancestral components most correlated with variations in vitamin B12 levels (Table 1). Significant correlations were observed for 51 (of 90 vitamin B12 related genes) gene regions with negative signals for both AFR and AMR and positive for EUR (Fig. 1b), consistent with local correlations previously observed. The number of gene regions correlated with AFR, AMR and EUR ancestries were 17, 28, and 39, respectively.

**Table 1:** Correlation between the local ancestry components AFR, AMR and EUR with baseline vitamin B12 levels per each 90 B12-related gene with *p*_perm_ < 0.1.

Gene symbol	AFR		AMR		EUR	
	r	*p*_perm_	r	*p*_perm_	r	*p*_perm_
ACOT7	− 0.08	2.9E−01	− 0.11	1.7E−01	0.13	**8.28E−02**
HES2	− 0.08	2.9E−01	− 0.11	1.6E−01	0.13	**8.23E−02**
UBIAD1	− 0.09	2.7E−01	− 0.11	1.6E−01	0.13	**8.71E−02**
MTHFR	− 0.09	2.5E−01	− 0.11	1.6E−01	0.13	**8.94E−02**
MMACHC	− 0.10	2.2E−01	− 0.08	3.2E−01	0.14	**7.29E−02**
SCP2	− 0.11	1.6E−01	− 0.05	5.1E−01	0.10	1.88E−01
CTH	0.02	8.5E−01	− 0.05	5.0E−01	0.05	5.42E−01
F3	− 0.05	5.2E−01	− 0.09	2.4E−01	0.06	4.34E−01
SARS	− 0.08	3.0E−01	− 0.05	5.0E−01	0.17	**2.74E−02**
CRP	− 0.15	**4.8E−02**	− 0.01	8.6E−01	0.07	3.77E−01
LIN9	− 0.10	2.2E−01	− 0.05	4.9E−01	0.06	4.78E−01
MTR	− 0.01	8.5E−01	− 0.11	1.6E−01	0.09	2.64E−01
THUMPD2	− 0.12	1.3E−01	− 0.01	9.3E−01	0.12	1.20E−01
TXNDC9	− 0.05	4.8E−01	− 0.07	3.9E−01	0.09	2.51E−01
MMADHC	0.00	9.8E−01	− 0.15	**5.4E−02**	0.14	**8.03E−02**
LRP2	− 0.13	**8.7E−02**	− 0.05	5.2E−01	0.09	2.59E−01
HAT1	− 0.16	**3.3E−02**	− 0.06	4.5E−01	0.13	**8.63E−02**
METAP1D	− 0.17	**3.2E−02**	− 0.06	4.6E−01	0.13	**8.70E−02**
CASR	− 0.11	1.4E−01	− 0.08	2.9E−01	0.14	**6.72E−02**
ALDH1L1	− 0.06	4.7E−01	− 0.11	1.6E−01	0.10	2.14E−01
TF	0.00	9.8E−01	− 0.18	**1.9E−02**	0.06	4.67E−01
CP	− 0.05	5.0E−01	− 0.17	**2.6E−02**	0.16	**4.04E−02**
LXN	− 0.02	7.7E−01	− 0.18	**1.9E−02**	0.14	**7.70E−02**
TFRC	− 0.16	**4.1E−02**	− 0.04	6.0E−01	0.16	**3.83E−02**
CSN1S1	0.03	6.9E−01	− 0.04	6.3E−01	0.05	4.81E−01
HTN3	0.03	6.9E−01	− 0.04	6.3E−01	0.05	4.95E−01
ALB	0.04	6.3E−01	− 0.06	4.1E−01	0.03	6.78E−01
METAP1	− 0.07	3.6E−01	0.06	4.0E−01	0.07	3.80E−01
GYPA	− 0.10	2.1E−01	0.01	8.6E−01	0.16	**3.34E−02**
MMAA	− 0.04	5.6E−01	0.02	7.9E−01	0.15	**5.92E−02**
MTRR	− 0.04	6.4E−01	− 0.13	1.0E−01	0.10	2.20E−01
PRELID2	− 0.05	5.3E−01	− 0.06	4.1E−01	0.10	2.03E−01
HRH2	− 0.04	5.8E−01	− 0.10	2.0E−01	0.10	1.91E−01
ADAMTS2	− 0.03	6.6E−01	− 0.16	**4.2E−02**	0.09	2.23E−01
PGC	− 0.10	1.9E−01	− 0.09	2.5E−01	0.14	**6.51E−02**
GNMT	− 0.12	1.2E−01	− 0.08	2.8E−01	0.13	**8.48E−02**
MUT	− 0.15	**5.7E−02**	− 0.08	3.0E−01	0.15	**4.51E−02**
LMBRD1	− 0.11	1.7E−01	− 0.08	2.9E−01	0.15	**4.99E−02**
PON1	− 0.04	6.2E−01	− 0.08	2.9E−01	0.05	4.89E−01
GGH	0.00	9.6E−01	− 0.11	1.6E−01	0.09	2.44E−01
FPGS	− 0.12	1.1E−01	− 0.19	**1.2E−02**	0.14	**6.41E−02**
SLC27A4	− 0.13	**9.1E−02**	− 0.20	**9.7E−03**	0.15	**6.02E−02**
CUBN	− 0.08	3.1E−01	− 0.15	**5.1E−02**	0.16	**3.75E−02**
TRDMT1	− 0.08	3.1E−01	− 0.15	**4.9E−02**	0.16	**4.37E−02**
PANK1	− 0.13	**9.2E−02**	− 0.15	**4.6E−02**	0.17	**2.64E−02**
PNLIP	− 0.14	**6.9E−02**	− 0.16	**4.2E−02**	0.17	**2.47E−02**
IFITM2	− 0.08	3.2E−01	− 0.14	**7.1E−02**	0.10	2.17E−01
HBE1	− 0.06	4.1E−01	− 0.11	1.6E−01	0.08	3.03E−01
GIF	− 0.19	**1.2E−02**	− 0.20	**1.1E−02**	0.24	**1.86E−03**
TCN1	− 0.19	**1.2E−02**	− 0.20	**1.1E−02**	0.24	**1.91E−03**
MS4A3	− 0.19	**1.3E−02**	− 0.20	**9.9E−03**	0.24	**1.71E−03**
PGA3	− 0.14	**6.1E−02**	− 0.21	**6.6E−03**	0.21	**6.07E−03**
PGA4	− 0.14	**6.4E−02**	− 0.21	**6.6E−03**	0.21	**5.85E−03**
FADS1	− 0.13	**8.2E−02**	− 0.21	**6.0E−03**	0.21	**6.93E−−03**
FTH1	− 0.12	1.1E−01	− 0.21	**6.2E−03**	0.20	**9.70E−03**
CBL	− 0.02	8.0E−01	− 0.25	**1.2E−03**	0.14	**7.59E−02**
CD4	− 0.02	7.5E−01	− 0.04	6.2E−01	0.12	1.13E−01
CS	− 0.10	2.0E−01	0.03	7.2E−01	0.09	2.45E−01
SHMT2	− 0.10	1.9E−01	0.03	7.0E−01	0.08	3.05E−01
METAP2	− 0.11	1.4E−01	− 0.02	7.9E−01	0.14	**6.19E−02**
MMAB	− 0.05	5.6E−01	0.06	4.7E−01	0.03	7.14E−01
MVK	− 0.05	5.6E−01	0.06	4.7E−01	0.03	7.14E−01
SDS	− 0.05	5.2E−01	0.08	3.1E−01	0.02	7.99E−01
ATP12A	− 0.04	6.2E−01	− 0.03	6.5E−01	0.05	5.01E−01
CLYBL	− 0.13	**8.7E−02**	− 0.04	6.3E−01	0.12	1.13E−01
ATP4B	− 0.08	3.2E−01	− 0.09	2.5E−01	0.13	**8.22E−02**
ABCD4	0.00	1.0E+00	− 0.12	1.1E−01	0.02	8.40E−01
AMN	0.01	9.5E−01	− 0.05	5.1E−01	− 0.01	9.28E−01
TGM5	− 0.12	1.3E−01	− 0.06	4.3E−01	0.16	**4.19E−02**
DUT	− 0.13	**8.4E−02**	− 0.10	1.9E−01	0.15	**4.60E−02**
NDUFAB1	− 0.10	1.9E−01	0.00	9.5E−01	0.10	2.02E−01
HP	− 0.08	3.2E−01	0.00	9.9E−01	0.06	4.35E−01
HPR	− 0.08	3.2E−01	0.00	9.9E−01	0.06	4.35E−01
PEMT	0.02	8.1E−01	− 0.12	1.1E−01	0.11	1.72E−01
TNFAIP1	0.01	8.9E−01	− 0.13	**8.2E−02**	0.10	1.95E−01
GAST	0.08	3.1E−01	− 0.14	**7.6E−02**	0.10	1.83E−01
ACLY	0.08	3.0E−01	− 0.14	**7.8E−02**	0.10	1.89E−01
MMD	0.01	8.5E−01	− 0.12	1.2E−01	0.12	1.30E−01
TYMS	− 0.12	1.4E−01	− 0.02	7.7E−01	0.11	1.44E−01
MBP	− 0.03	6.6E−01	− 0.02	7.9E−01	0.04	5.76E−01
FUT6	− 0.03	6.9E−01	− 0.15	**4.8E−02**	0.11	1.40E−01
CD320	− 0.07	3.9E−01	− 0.10	2.1E−01	0.07	3.46E−01
SLC27A1	− 0.10	2.1E−01	− 0.10	1.9E−01	0.11	1.68E−01
ATP4A	− 0.03	6.6E−01	− 0.12	1.2E−01	0.05	5.54E−01
CD79A	0.00	9.7E−01	− 0.13	**8.4E−02**	0.08	3.29E−01
FUT2	− 0.04	6.1E−01	− 0.14	**6.8E−02**	0.15	**4.82E−02**
FTL	− 0.05	5.1E−01	− 0.14	**7.4E−02**	0.16	**3.36E−02**
NTSR1	0.06	4.4E−01	− 0.17	**2.6E−02**	0.00	9.95E−01
CBS	0.10	1.9E−01	− 0.12	1.3E−01	0.01	8.79E−01
TCN2	− 0.02	7.5E−01	− 0.04	6.4E−01	0.04	6.45E−01

r: Spearman correlation.

Bold values denote *p*_*per*_ < 0.1.

### Association between variants in the set of 90 B12-related genes and B12 levels

Of a total of 3,406,465 SNP variants from genotype and exome analysis, 6699 were found to be present in the 90 B12-related genes. These SNPs were grouped in 1971 clumps where the most significant SNPs from GEE were considered as the representative SNP of the clump (SNP_ref_). After multiple testing correction of *p*-values obtained from GEE for these 1971 SNP_ref_, thirty-six variants in 26 genes were associated with the B12 baseline vitamin levels at *p*_*adj*_ < 0.1 (Table 2). The most significant SNP_ref_ (kgp6592612, *p*_adj_ = 4.22 × 10^–^^12^) was in FUT6 gene (Chr19) which showed a negative association with vitamin B12 levels. Fifteen SNP_ref_ showed a negative association with phenotype, while 21 showed a positive association. Among the SNP_ref_, 16 had no SNP in LD, whereas 6 had one SNP in LD, 3 had two SNP in LD and 11 had 3 or more SNPs in LD.

**Table 2:** Statistically significant GEE association between 36 SNPs in the 26-related vitamin B12 genes and B12 basal levels^a^.

SNP_ref_	Major	Minor	MAF	Chr	Position (bp)	Gene symbol	GEE Association between SNP and vitamin B12 levels	
							*β*_*Assoc*_	*p*_adj_
rs2182598	A	T	0.0119	1	109,766,687	SARS	0.39	1.0E−02
rs12081406	A	G	0.01786	1	109,778,391	SARS	0.45	6.1E−02
rs2808631	T	C	0.02976	1	159,682,036	CRP	− 0.62	4.0E−03
rs10925263	A	G	0.1131	1	237,063,748	MTR	0.31	5.5E−02
rs138583897	T	G	0.0119	2	170,061,860	LRP2	0.91	7.1E−03
rs74791051	A	G	0.01786	2	170,085,774	LRP2	− 0.44	2.2E−03
rs144147038	C	T	0.0119	2	170,145,661	LRP2	− 0.49	1.9E−02
rs1800249	C	A	0.04762	3	133,464,794	TF	0.42	4.7E−02
rs7633232	G	C	0.02083	3	133,475,614	TF	0.52	6.1E−02
rs78536844	G	A	0.01488	3	158,388,161	LXN	0.79	9.0E−02
rs1220841	G	A	0.03869	4	70,795,699	CSN1S1	0.49	5.4E−03
rs1529038	A	G	0.1637	4	70,795,920	CSN1S1	− 0.33	1.5E−02
rs10489132	A	G	0.03571	4	99,950,417	METAP1	− 0.41	5.5E−02
rs1304811491	A	G	0.0119	5	178,768,430	ADAMTS2	0.55	5.5E−02
rs141206548	T	C	0.01488	6	70,408,871	LMBRD1	− 0.54	4.6E−02
rs116157801	G	A	0.02381	9	130,569,812	FPGS	0.40	8.2E−02
rs144213212	A	G	0.05357	9	131,118,554	SLC27A4	0.30	9.1E−02
rs117412228	G	A	0.02083	10	17,046,009	CUBN	0.51	3.5E−02
rs11254386	G	C	0.0119	10	17,171,907	CUBN	0.27	8.9E−03
rs116286548	A	G	0.0119	11	59,630,345	TCN1	− 0.77	4.7E−02
rs28989521	A	G	0.04167	12	6,898,084	CD4	− 0.44	4.0E−02
rs72648013	G	A	0.02381	12	110,025,617	MVK	0.45	5.4E−03
rs41288280	G	A	0.0119	13	25,272,833	ATP12A	− 0.91	1.3E−03
rs721922	A	G	0.1339	13	100,462,812	CLYBL	− 0.30	3.8E−02
rs148126159	G	A	0.01488	14	74,752,605	ABCD4	0.71	4.2E−06
rs79752143	G	A	0.02679	16	23,606,959	NDUFAB1	0.55	9.8E−04
rs147671744	G	A	0.02083	17	17,460,540	PEMT	− 0.46	6.1E−02
rs8083543	C	T	0.02083	18	74,726,712	MBP	0.43	6.1E−02
rs116910911	G	A	0.02083	18	74,768,653	MBP	0.46	4.7E−02
rs75243301	A	G	0.0119	19	5,841,156	FUT6	− 0.32	4.4E−02
rs6090388	G	A	381	20	61,336,359	NTSR1	0.20	7.3E−02
rs34864360	C	T	0.01488	20	61,386,225	NTSR1	0.37	1.1E−02
rs45613333	G	A	0.0119	20	61,391,514	NTSR1	− 0.67	4.3E−04
rs111386779	C	T	0.02083	21	44,473,450	CBS	0.44	1.4E−02
rs150181241	C	T	0.01488	21	44,483,279	CBS	− 0.47	6.1E−02
brz:22:31023366:AG	A	G	0.0119	22	31,023,366	TCN2	− 1.02	2.9E−04

^a^The SNPs were filtered using GEE (generalized estimated equation) association test with *p*_adj_ significance < 0.1. The covariates were sex, BMI, age, total HEI and mean ancestry genetic ancestry. The data includes the names of SNPs and their respective coordinates, with *brz* prefix indicating the SNPs extracted from exome. MAF: Minor Allele Frequency; Chr: Chromosome. The alleles here are those that are defined by Illumina genotyping or exome data and the alleles noted for some SNPs differ from dbSNP. These divergences occur because the annotation in the dbSNP can be for the complementary base and vice versa for the Illumina chip.

### Polygenic risk score construction

**Figure 2:**
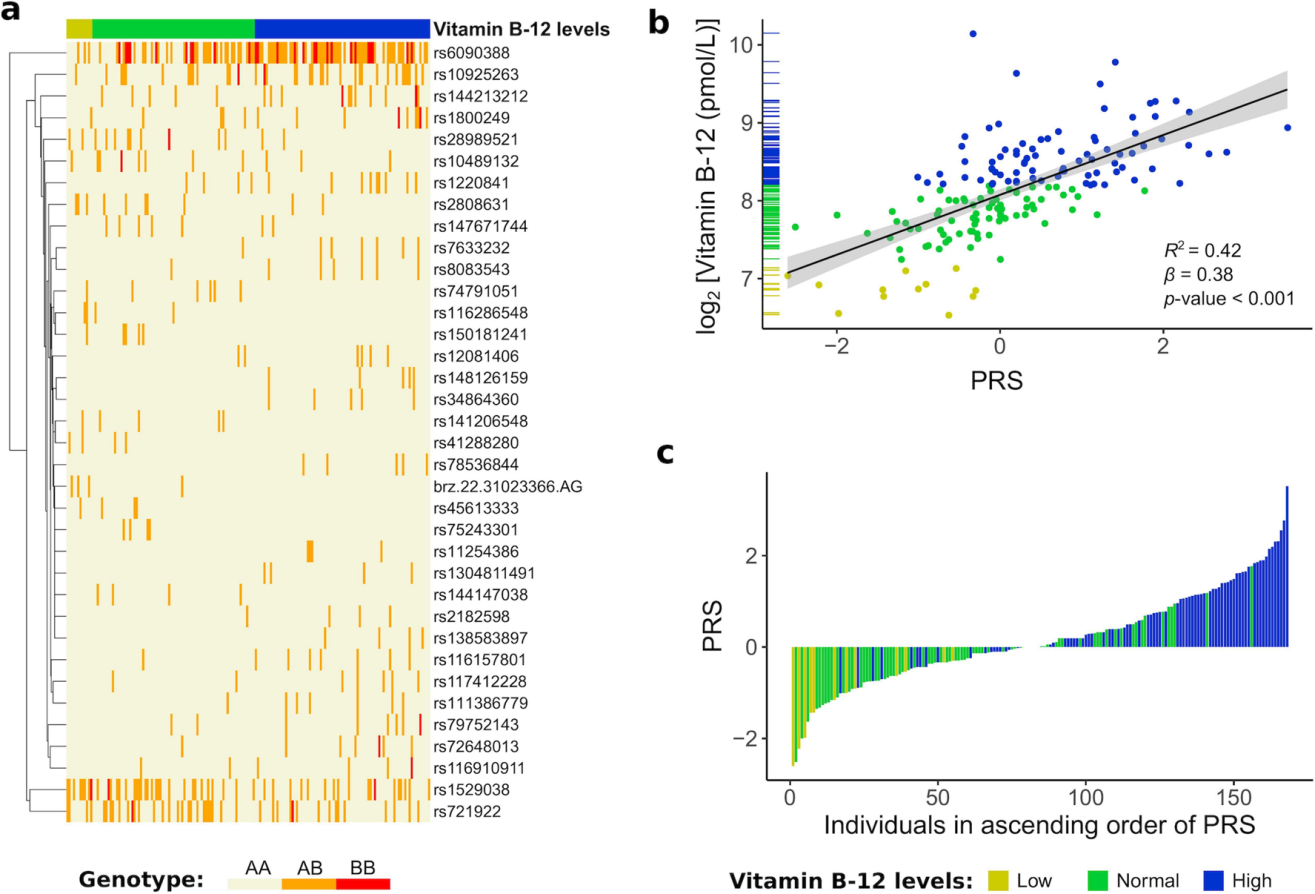
Heatmap of 36 variants associated with baseline vitamin B12 levels. BB is minor allele (**a**). PRS regression to vitamin B12 levels (**b**). Individuals in ascending order of PRS (**c**).

Thirty-six SNP_ref_ from the additive model (Fig. 2a) were used to calculate a polygenic risk score. PRS explained 42% of phenotype (Fig. 2b). Figure 2c shows individuals in ascending order of PRS.

**Figure 3:**
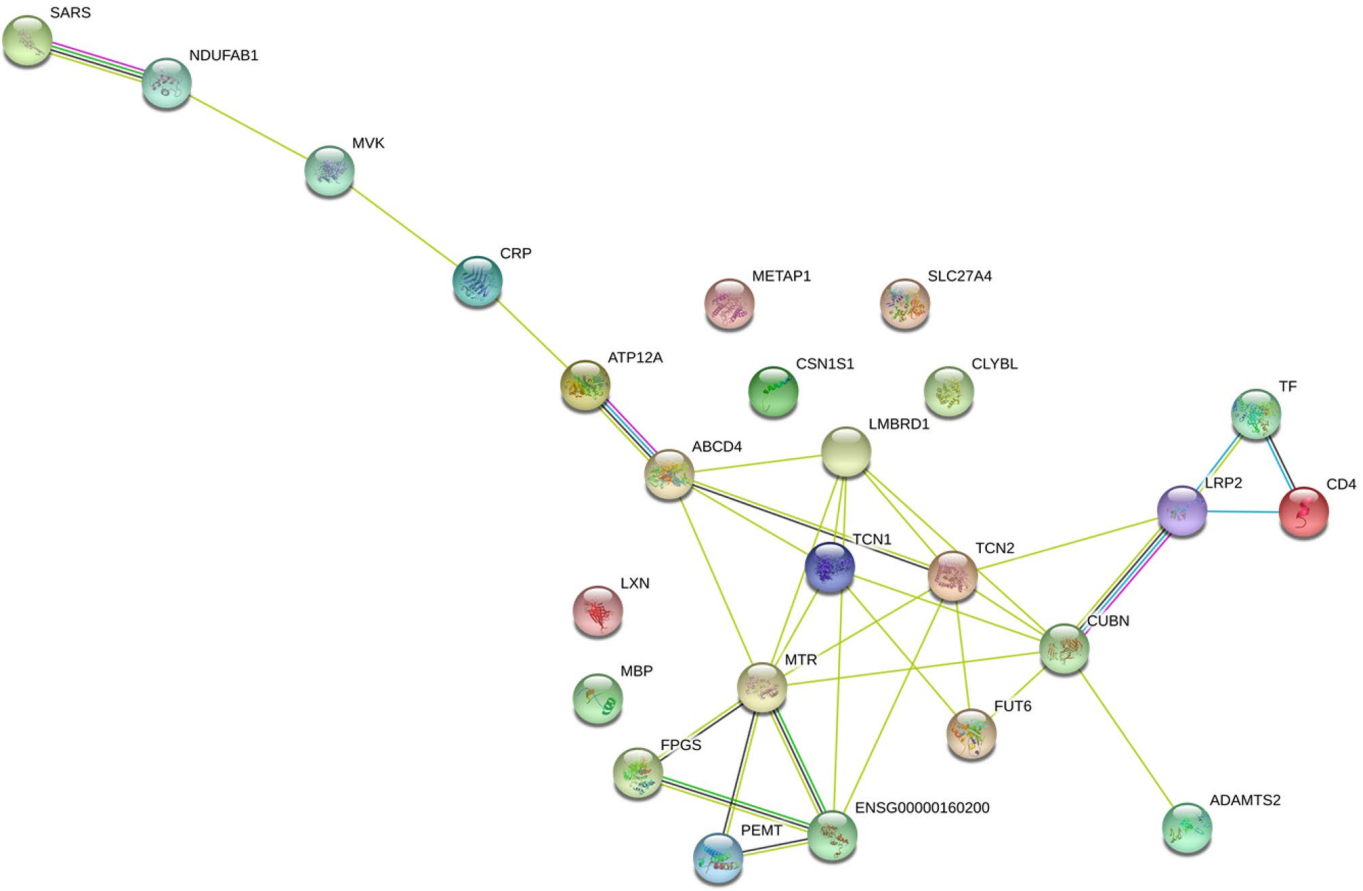
26 genes in a protein–protein association network for vitamin B12, using String.

Although all the genes selected for this study were all associated with vitamin B12 metabolism, transport, or biochemistry, we used String version 11.0^63^ to visualize protein interactions among the 26 genes used to constitute the PRS. A protein–protein enrichment *p*-value lower than 1.0 × 10^–^^16^ was found for the whole network (Fig. 3; Additional file 3: Excel files Supplement Material). Interacting proteins in the network were associated with many biological processes, including direct cobalamin metabolic processes (with FDR *p*-value equal to 6.74 × 10^–^^10^ for *ABCD4, CUBN, LMBRD1, MTR, TCN1, TCN2*) and cobalamin transport (*p*-value 1.73 × 10^–^^7^ for *CUBN, LMBRD1, TCN1, TCN2*), as expected. However, less significant but nonetheless interesting interactions suggest that vitamin B12 levels may impact lipid metabolism (*p*-value 0.0053 for *CUBN, MVK, NDUFAB1, PEMT, SLC27A4*) and coated pit processes (*p*-value 0.024 for *CUBN, LRP2*). During the first steps of the vesicle-mediated membrane transport, coated pits are internalized to form coated vesicles which transport proteins between distinct membrane-bound organelles. Vitamin B12 levels may also have a role in vitamin D metabolic process primarily through the low-density lipoprotein receptor-related protein 2 (*p*-value 0.009 for *CUBN, LRP2*) and in alpha-amino acid metabolic process (*p*-value 0.0005 for *CBS, FPGS, MTR, PEMT, SARS*).

**Figure 4:**
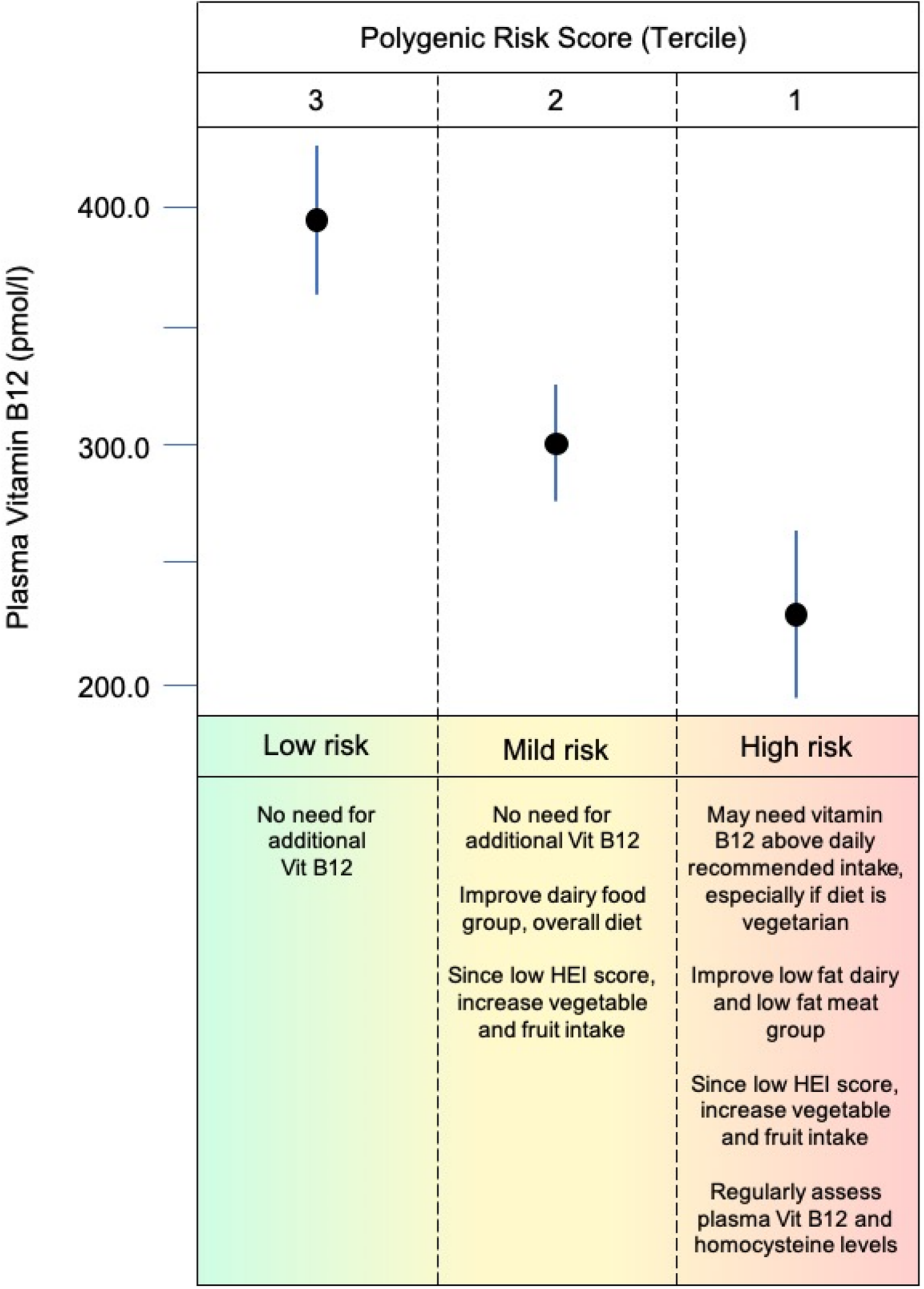
Hypothetical proposed nutritional counseling for vitamin B12 intake based on PRS terciles, nutritional and demographic data.

We stratified PRS of individuals into terciles to assess its association with age, sex, BMI, total HEI, meat and milk intake (HEI components), socioeconomic status and levels of vitamin B12, tHcy, folate, pyridoxal and riboflavin, in order to build a hypothetical nutritional counseling diagram (Table 3; Fig. 4). In the total studied individuals, only 7% (n = 12) had low vitamin B12 levels, 45% (75) had normal values and 48% (81) had high levels.

**Table 3:** Characteristics of individuals according to terciles of polygenic risk score.

	Continuous data (mean ± SD)			
	PRS-T1 (− 2.60 to − 0.30)	PRS-T2 (− 0.30 to 0.41)	PRS-T3 (0.43 to 3.52)	*P*_KW*_
n	56	56	56	–
Age (year)	11.32 ± 1.08	11.41 ± 1.11	11.66 ± 1.08	0.27
BMI (kg/m^2^)	19.80 ± 4.50	20.66 ± 4.11	19.66 ± 4.48	0.22
HEI (score)	54.42 ± 10.69	54.20 ± 7.87	53.03 ± 11.82	0.53
MilkGroup (score)	5.06 ± 3.72	6.49 ± 3.34	5.10 ± 3.82	0.06
MeatGroup (score)	8.90 ± 2.51	9.30 ± 1.60	8.68 ± 2.45	0.54
Vitamin B12 levels (pmol/L)	227.04 ± 145.38	303.23 ± 101.07	396.26 ± 129.81	**< 0.01**
n	53	56	56	
Folate levels (nmol/L)	11.26 ± 5.96	11.47 ± 4.55	10.76 ± 4.12	0.81
n	54	55	56	
Pyridoxal levels (nmol/L)	7.81 ± 3.27	7.46 ± 2.58	9.36 ± 7.25	0.07
n	49	54	51	
Riboflavin levels (nmol/L)	13.91 ± 11.43	13.26 ± 8.58	15.66 ± 10.30	0.16
n	49	47	51	
tHcy levels (µmol/L)	2.85 ± 0.71	2.92 ± 0.90	2.50 ± 0.67	**0.02**

	Categorical data (%)			
	PRS-T1	PRS-T2	PRS-T3	*p-value*_Chi2/Fisher*_
**Socioeconomic status (%) **				0.87
A1	–	–	–	
A2	37.5	12.5	50.0	
B1	31.8	40.9	27.3	
B2	28.6	39.3	32.1	
C1	33.3	31.2	35.4	
C2	44.0	28.0	28.0	
D	33.3	22.2	44.4	
E	–	–	–	
**Sex (%) 0.94**				0.94
Female	26.4	33.0	40.7	
Male	41.6	33.8	24.7	

*p values were calculated by Kruskal–Wallis (*P*_KW_), Chi-square (*P*_Chi2_) or Fisher test. Bold values denote *p* < 0.05. PRS-T: polygenic risk score tercile. A1 and A2: higher income. B1 and B2: medium income. C1, C2, D and E: lower income.

## Discussion

The development of broad public health strategies for disease prevention requires the identification of risk factors. Vitamin B12 insufficiency may be a risk factor that contributes to the substantial burden of diseases in the general population. Literature and database searches identified 90 genes related with vitamin B12 basal levels. We determined the genetic ancestry of each of these genes in individuals in the study population: thirty-nine genes were positively correlated with EUR ancestry, twenty-eight genes with Amerindian ancestry, and seventeen genes in regions of AFR ancestry. Brazilians share EUR, AMR and AFR ancestries^65^ along with minor contributions from many other geographic areas. Our results emphasize the contribution of individual genetic ancestry as a predictor for a clinically relevant phenotype and perhaps disease onset^21^.

Individual SNPs may contribute only modestly to a phenotype but in combination could explain a significant portion of the variation in phenotype incidence in the general population^45^. From the set of 36 SNPs associated with vitamin B12, FUT6, the gene encoding for a fucosyltransferase (an enzyme that allows for the addition of fucose to oligosaccharides) was the most significant. Polymorphisms in FUT genes have been shown to influence the quantity and quality of Human Milk Oligosaccharides (HMOs) in breast milk, and HMOs are exerting a prebiotic as well as immunomodulatory effects on the infant gut^66^. A polymorphism in an enzyme of the same family, FUT2, has been associated to susceptibility to *Heliobacter pylori* infection in Amazonian children^67^. Thus, bacterial overgrowth may be one of causes leading to vitamin B12 deficiency.

Taken together, the combined set of 36 SNPs statistically associated with vitamin B12 levels in this study were used to create a PRS to predict vitamin B12 levels. The PRS explained 42% of vitamin basal level variation, extending the results reported in Mathias et al.^17^. The heritability of B12 levels was estimated to be 59% in a study using monozygotic and dizygotic twins, indicating that the magnitude of genetic influence on vitamin B12 levels may be considerable^68^. Other genetic studies also indicate that vitamin B12 status is a multifactorial trait, where several single-nucleotide polymorphisms (SNPs) in multiple genes interact with the environment to cause the altered B12 status^37,69^. Genetic variants may alter vitamin B12 tissue status by affecting the proteins involved in vitamin B12 absorption, cellular uptake and intracellular metabolism^37,70^. The underlying genetic architecture for levels of other vitamins have not been characterized. The use of PRS are becoming clinically highly useful^71,72^ and the SNPs identified in this study may contribute to variations in B12 levels.

We used a middle out approach for choosing genes involved in vitamin B12 metabolism. Middle out is an emerging approach that uses a predetermined subset of high-dimensional data that are limited to a system of interest^44^.

Multiple variants in a subset of these genes collectively explained more of the variation of vitamin level than could be explained by a single variant. Many recent Mendelian randomization investigations of complex traits (such as blood pressure, BMI, or blood lipids) have used multiple variants^73,74^ and the same can apply to vitamin levels. This middle out approach and subsequent analysis identified 26 genes encoding 36 SNPs associated with B12 levels. Functional interactions among these genes were analyzed using String^63^. Interactions among *LMBRD1* (lysosomal cobalamin transport escort protein), *CUBN* (cubilin), *TCN1* (transcobalamin 1), *TCN2* (transcobalamin 2), and ABCD4 (lysosomal cobalamin transporter *ABCD4*) were expected since they are involved in the transport or in the lysosomal release of vitamin B12 into the cytoplasm. Disruptions in the transport machinery of vitamin B12 have then consequences on its bioavailability. While mutations in *TNC2* may lead to anemia and profound neurological disorders, *TNC2* polymorphisms may lead to differential blood concentration of transcobalamin^29^. Given the link between vitamin B12 and the methionine cycle, the genes *CUBN*, *ABCD4* with *MTR* (exosome RNA helicase) were identified, as these participate in reactions that catalyze the transfer of a methyl group from methyl-cobalamin to homocysteine^63^. Interactions in a smaller subset of the identified genes are involved in lipid metabolism, which links to another metabolic role of vitamin B12, the involvement in mitochondrial metabolism: (i) *NDUFAB1* (mitochondrial acyl carrier) is an acyl carrier protein of the growing fatty acid chain in fatty acid biosynthesis, (ii) *MVK* (mevalonate kinase) is a regulatory site in cholesterol biosynthetic pathway, (iii) *PEMT* (phosphatidylethanolamine *N*-methyltransferase) catalyzes the three sequential steps of the methylation pathway involving phosphatidylethanoamine (PE), phosphatidylmonomethylethanolaimne (PMME), phosphatidyldimethylethanolamine (PDME), phosphatidylcholine (PC), (iv) *SLC27A4* (long-chain fatty acid transport protein 4) is a long-chain fatty acid transport protein, and (v) *CUBN* is a cotransporter lipoprotein metabolism^63^. In addition, megalin (*LRP2*, low-density lipoprotein receptor-related protein 2) acts together with *CUBN* to mediate endocytosis of high-density lipoproteins^63^. Intestinal vitamin B12 malabsorption is found in the rare disease Imerslund–Gräsbeck syndrome, that entails mutations in cubilin and amnionless (*AMN*)^29^. These potential interactions are consistent with a system view of metabolic processes in that genes involved in B12 metabolism also participate in other physiological processes.

Torkamani et al.^46^ proposed that PRS can be used for three classes of interventions: PRS-informed therapeutic intervention (the part that PRS can play in the selection of interventions to treat or prevent disease); PRS-informed disease screening (the role that PRS can have in the decision to initiate and the interpretation of disease screens); and PRS-informed life planning (the personal utility that PRSs can provide, even in the absence of preventive actions). Based on this categorization, we propose a hypothetical nutritional counseling based on PRS terciles, nutritional, and demographic data assuming that PRS can play a role in helping to screen, to prevent insufficiencies/deficiencies or to select interventions^46^. In addition, nutritional counseling based on PRS and considering cofounding variables may individualize management of vitamin B12 recommendation and personalize health care.

## Strength of the study

From 6699 SNPs analyzed by genotyping and exome analysis within the 90 B12 related genes, 36 were associated with vitamin B12 levels after correcting for multiple testing and adjusting for covariates such as age, sex, BMI, quality of diet and genetic ancestry, as suggested by Goetz et al.^21^. Including the covariates may have increased statistical power. Some studies also adjust for other characteristics, including directly measured traits (such as age and sex)^74^. Association between a genetic variant and outcome may also be confounded by hidden population structure. This can be addressed by adjusting for genetic ancestry^74^ as done in this study. The 36 SNPs used to create the PRS may improve the accuracy and reliability of it.

Moreover, the resulting significant SNPs obtained from GEE were employed to clump (i.e., to create haplotypes) of variants within a window of 250 kb that are in LD in order to find the more significant SNPs for each clump. Associations accounting for LD have been shown to improve the performance of PRS models in some settings^75^.

Others have found that any significant association identified between genetic ancestry and disease or clinical condition is greatly attenuated after controlling for socioeconomic status and others environmental factors^21,76,77^. In the current study, socioeconomic status was similar among the PRS terciles.

Our results are consistent with the concept^21^ that genetic background impacts clinically relevant intermediate phenotypes, one of the first examples of which was the effect of ethnicity on a haplotype containing LTA4H on myocardial risk^78^. These types of results should motivate further research in the field of nutrition.

## Limitations of the study

One-carbon metabolism is influenced by a variety of nutrients that interact and can compensate for one another when there is a deficiency of a single nutrient. The potential role of other micronutrients and metabolites in one-carbon metabolism, such as homocysteine, folate, riboflavin, and pyridoxine need to be examined, as they may influence both vitamin B12 metabolism^26,79^ and, consequently, the power of PRS. Although we did not adjust the PRS for these metabolites, the categorization of individuals into terciles showed no statistical difference for those B-vitamins. tHcy levels in RBC were higher in the first tercile but still within normal ranges.

We evaluated plasma B12 levels. Serum or plasma B12 measurement has advantages of (i) being widely available and inexpensive, (ii) not greatly affected by recent intake, (iii) concentrations within an individual are relatively constant, (iv) there is no need for fasting before sample collection and (v) is not influenced by age or infection, (vi) but are not overly-sensitive or -specific which would produce false positive and negative diagnoses^80^. A second indicator, such as serum methylmalonic acid (MMA) and/or holo-transcobalamin, should be assessed to improve problems with sensitivity and specificity^81^, which was not evaluated in the present study. The cut-off for vitamin B12 deficiency chosen was < 148 pmol/L, which corroborates other studies^32,80–83^. However, no consensus exists for a definition of a cut-off point low vitamin B12 levels in children and adolescents^30,35,80^.

The identification of genetic variants that contribute to more complex multifactorial conditions with a polygenic risk score may not be useful for predicting phenotype incidence rates in another population because the SNPs identified in one population may have different frequencies in a different population^21^. Therefore, PRS must be tested in populations with different genetic ancestries. In addition, greater uncertainty exists when using PRS because some of these SNPs may only be correlated with the causal genetic factor or factors. This may reduce the generalizability of PRS risk estimates in populations beyond the population studied. Missing heritability, the unknown component of genetic risk, is another source of uncertainty specific to polygenic risk estimates^46^.

The present study was based on small sample size for genetic analysis and considered only 3 major globally admixed populations. While the p-values for these SNPs were above the typical GWAS threshold of 5 × 10^–^^8^, the strategy was to identify the combinations of low effect SNPs that could be associated with B12 levels. Despite the inherent limitations of GWAS-PRS that are associated with sample size, the middle out approach used here investigated a possible PRS constructed on a given set of known phenotype related genes/SNPs data^45^. The ability to adjust statistical correlations for covariates, test other phenotypic variables (e.g., tHcy in RBC), and develop nutritional counseling strategies was a direct result of the depth of phenotypic analysis^17^ that was conducted in this study, a trade-off for larger study populations usually used for genetic studies.

The results presented should be validated in other studies and might serve as a guide for future studies of larger numbers of participants. We propose an approach to motivate further research and help move the biomedical research community towards greater sensitivity to global issues in population health.

## Conclusion and future considerations

The basic components of disease risk are genetic susceptibility, environmental exposures, and lifestyle factors and these factors are often considered separately. Our study is consistent with the concept that genetic background impacts vitamin levels. The middle out approach used here investigated a possible PRS to predict vitamin B12 levels that explained 42% of vitamin basal level variation. Its performance should be evaluated in an independent sample, or using cross validation techniques. In the near future, large scale sequencing and imputation-based association studies will provide additional data and a more comprehensive assessment of the role of rare and low frequency variants that may confer more moderate risk to diseases^45^. Improvements in models through the incorporation of polygenic risk and possibly other predictive factors, may identify individuals at different levels of risk for developing diseases. Such data could be translated into improvements in primary and secondary prevention by tailoring interventions according to risk^45^ such as those within the tercile of genetic risk of low levels of vitamin B12 that are at increased risk. These types of considerations would strengthen the field of nutrition by creating more effective applications of nutrition to help public health.

## Supplementary Information

Supplementary Information 1.

Supplementary Information 2.

Supplementary Information 3.

## Data Availability

The datasets supporting the conclusions of this article are included within the article and its additional files. The raw data are available from the corresponding author on reasonable request.

## Abbreviations

AFR: African
AMR: Native American
BMI: Body mass index
Chr: Chromosome
ELAI: Efficient Local Ancestry Inference
EUR: European
FDR: False Discovery Rate
GEE: Generalized estimating equation
GRCh37: Genome Reference Consortium Human genome build 37
GWAS: Genome-wide association study
HCFMRP-USP: Ribeirão Preto Medical School Hospital
HEI: Healthy eating index
HGDP: Human Genome Diversity Project
LC–MS/MS: Liquid chromatography tandem mass spectrometry
LD: Linkage disequilibrium
LTA4H: Leukotriene A4 hydrolase
MAF: Minor allele frequency
PRS: Polygenic risk score
PRS-T: Polygenic risk score tercile
QC: Quality control
SD: Standard deviation
SNP: Single nucleotide polymorphism
RBC: Red blood cell
WHO: World Health Organization
