# Supplementary material for: Contribution of genetic ancestry and polygenic risk score in meeting vitamin B12 needs in healthy Brazilian children and adolescents
Source: Sci Rep. 2021 Jun 7;11:11992. doi: 10.1038/s41598-021-91530-7 (PMC8184816; doi:10.1038/s41598-021-91530-7)
Supplement: Supplementary file 1 — Supplementary Information 1. [file 41598_2021_91530_MOESM1_ESM.docx]

**Contribution of Genetic Ancestry and Polygenic Risk Score in Meeting Vitamin B12 Needs in Healthy Brazilian Children and Adolescents**

Carlos Alessandro Fuzo^1^, Fábio da Veiga Ued^2^, Sofia Moco^3^, Ornella Cominetti^4^, Sylviane Métairon^4^, Solenn Pruvost^4^, Aline Charpagne^4**^, Jerome Carayol^4^, Raul Torrieri^5^, Wilson Araujo Silva Jr^6^, Patrick Descombes^4^, Jim Kaput^4,7^, Jacqueline Pontes Monteiro^2*^

^1^ Department of Clinical Analyses, Toxicology and Food Sciences, School of Pharmaceutics Sciences, University of São Paulo, Ribeirão Preto, Brazil

^2^ Department of Pediatrics and Department of Health Sciences, Ribeirão Preto Medical School, Nutrition and Metabolism Section, University of São Paulo, Ribeirão Preto, Brazil

^3^ Department of Chemistry and Pharmaceutical Sciences, Amsterdam Institute for Molecular and Life Sciences, Vrije Universiteite Amsterdam

^4^ Nestlé Research, Société des Produits Nestlé SA, EPFL Innovation Park, H, CH1015 Lausanne, Switzerland

^5^ Center for Medical Genomics, Ribeirão Preto Medical School Hospital, University of São Paulo, Ribeirão Preto, Brazil

^6^ Department of Genetics, Ribeirão Preto Medical School, University of São Paulo, Ribeirão Preto, Brazil

^7^ Vydiant, Folsom, CA, USA

* Corresponding author: Jacqueline Pontes Monteiro. Avenida Bandeirantes, 3900. Bairro Monte Alegre. Ribeirão Preto, SP, Brazil. Postal code: 14.040-900. Phone: +55(16) 991548893. e-mail: jacque160165@gmail.com

** Present address: Sophia Genetics, Campus Biotech, CH-1202 Geneva, Switzerland

**Additional file 1: Supplemental Figures**


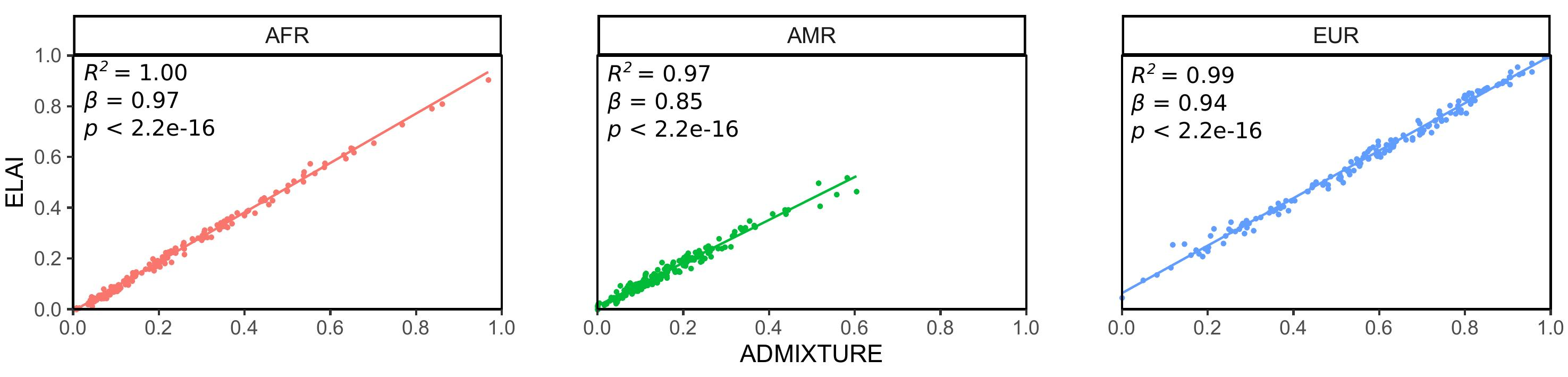


**Figure S1.** Linear regression for direct comparison between the mean local ancestry calculated with ELAI and global ancestry calculated with ADMIXTURE for components AFR, AMR and EUR of three-way admixture.


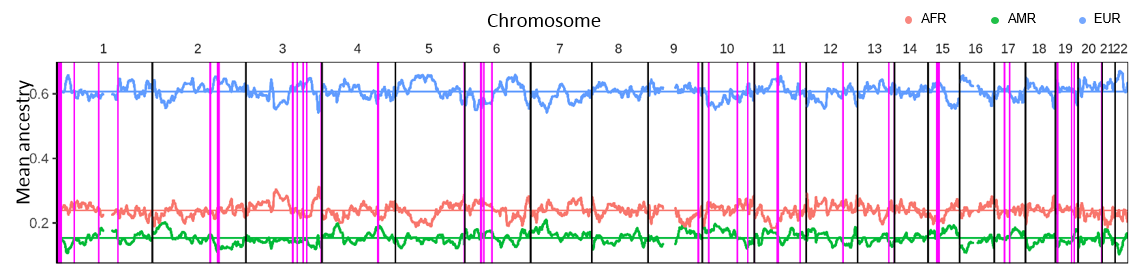


**Figure S2.** Mean ancestry along the chromosomes for the three ancestry components AFR, AMR and EUR. The vertical lines (magenta) represented the genes correlated with some ancestry component. The horizontal lines corresponded to genome wide mean ancestry for all children.
