# Supplementary material for: Contribution of genetic ancestry and polygenic risk score in meeting vitamin B12 needs in healthy Brazilian children and adolescents
Source: Sci Rep. 2021 Jun 7;11:11992. doi: 10.1038/s41598-021-91530-7 (PMC8184816; doi:10.1038/s41598-021-91530-7)
Supplement: Supplementary file 2 — Supplementary Information 2. [file 41598_2021_91530_MOESM2_ESM.docx]

**Contribution of Genetic Ancestry and Polygenic Risk Score in Meeting Vitamin B12 Needs in Healthy Brazilian Children and Adolescents**

Carlos Alessandro Fuzo^1^, Fábio da Veiga Ued^2^, Sofia Moco^3^, Ornella Cominetti^4^, Sylviane Métairon^4^, Solenn Pruvost^4^, Aline Charpagne^4**^, Jerome Carayol^4^, Raul Torrieri^5^, Wilson Araujo Silva Jr^6^, Patrick Descombes^4^, Jim Kaput^4,7^, Jacqueline Pontes Monteiro^2*^

^1^ Department of Clinical Analyses, Toxicology and Food Sciences, School of Pharmaceutics Sciences, University of São Paulo, Ribeirão Preto, Brazil

^2^ Department of Pediatrics and Department of Health Sciences, Ribeirão Preto Medical School, Nutrition and Metabolism Section, University of São Paulo, Ribeirão Preto, Brazil

^3^ Department of Chemistry and Pharmaceutical Sciences, Amsterdam Institute for Molecular and Life Sciences, Vrije Universiteite Amsterdam

^4^ Nestlé Research, Société des Produits Nestlé SA, EPFL Innovation Park, H, CH1015 Lausanne, Switzerland

^5^ Center for Medical Genomics, Ribeirão Preto Medical School Hospital, University of São Paulo, Ribeirão Preto, Brazil

^6^ Department of Genetics, Ribeirão Preto Medical School, University of São Paulo, Ribeirão Preto, Brazil

^7^ Vydiant, Folsom, CA, USA

* Corresponding author: Jacqueline Pontes Monteiro. Avenida Bandeirantes, 3900. Bairro Monte Alegre. Ribeirão Preto, SP, Brazil. Postal code: 14.040-900. Phone: +55(16) 991548893. e-mail: jacque160165@gmail.com

** Present address: Sophia Genetics, Campus Biotech, CH-1202 Geneva, Switzerland

**Additional File 2: Supplemental Tables**

**Table S1.** Ninety genes related to vitamin B12 levels and their coordinates in NCBI (37.3)^a^.

| Gene Symbol | Chromosome # | Start (bp) | End (bp) | Strand | Border(kb) |
| --- | --- | --- | --- | --- | --- |
| ACOT7 | 1 | 6322832 | 6458826 | - | 0 |
| HES2 | 1 | 6473792 | 6489724 | - | 0 |
| UBIAD1 | 1 | 11328255 | 11349991 | + | 23 |
| MTHFR | 1 | 11844287 | 11871160 | - | 7 |
| MMACHC | 1 | 45960856 | 45978239 | + | 55 |
| SCP2 | 1 | 53387901 | 53518789 | + | 0 |
| CTH | 1 | 70871901 | 70907034 | + | 0 |
| F3 | 1 | 94993232 | 95012413 | - | 0 |
| SARS | 1 | 109751515 | 109782304 | + | 2 |
| CRP | 1 | 159680579 | 159689396 | - | 9 |
| LIN9 | 1 | 226417350 | 226502789 | - | 12 |
| MTR | 1 | 236953581 | 237068781 | + | 2 |
| THUMPD2 | 2 | 39961700 | 40011416 | - | 9 |
| TXNDC9 | 2 | 99933987 | 99962155 | - | 75 |
| MMADHC | 2 | 150424647 | 150449330 | - | 13 |
| LRP2 | 2 | 169982119 | 170224123 | - | 0 |
| HAT1 | 2 | 172773935 | 172850100 | + | 23 |
| METAP1D | 2 | 172859804 | 172948658 | + | 0 |
| CASR | 3 | 121897530 | 122006850 | + | 0 |
| ALDH1L1 | 3 | 125820904 | 125905029 | - | 0 |
| TF | 3 | 133459977 | 133499350 | + | 0 |
| CP | 3 | 148878697 | 148944832 | - | 0 |
| LXN | 3 | 158382703 | 158395482 | - | 0 |
| TFRC | 3 | 195774655 | 195814032 | - | 3 |
| CSN1S1 | 4 | 70791799 | 70813788 | + | 1 |
| HTN3 | 4 | 70889130 | 70903755 | + | 26 |
| ALB | 4 | 74264972 | 74288629 | + | 47 |
| METAP1 | 4 | 99911788 | 99985460 | + | 3 |
| GYPA | 4 | 145028956 | 145066904 | - | 0 |
| MMAA | 4 | 146535540 | 146582687 | + | 48 |
| MTRR | 5 | 7846299 | 7902737 | + | 0 |
| PRELID2 | 5 | 145137082 | 145219899 | - | 0 |
| HRH2 | 5 | 175079847 | 175137739 | + | 0 |
| ADAMTS2 | 5 | 178536352 | 178777431 | - | 0 |
| PGC | 6 | 41702949 | 41720139 | - | 0 |
| GNMT | 6 | 42923500 | 42933118 | + | 38 |
| MUT | 6 | 49396573 | 49436041 | - | 24 |
| LMBRD1 | 6 | 70384141 | 70512049 | - | 0 |
| PON1 | 7 | 94926169 | 94958884 | - | 0 |
| GGH | 8 | 63926138 | 63956610 | - | 29 |
| FPGS | 9 | 130560137 | 130578299 | + | 2 |
| SLC27A4 | 9 | 131097839 | 131125249 | + | 8 |
| CUBN | 10 | 16864465 | 17176816 | - | 0 |
| TRDMT1 | 10 | 17178328 | 17249093 | - | 0 |
| PANK1 | 10 | 91337754 | 91410329 | - | 0 |
| PNLIP | 10 | 118300428 | 118328867 | + | 0 |
| IFITM2 | 11 | 303095 | 310910 | + | 3 |
| HBE1 | 11 | 5288080 | 5296373 | - | 7 |
| GIF | 11 | 59595246 | 59617974 | - | 0 |
| TCN1 | 11 | 59618781 | 59639041 | - | 15 |
| MS4A3 | 11 | 59819101 | 59840088 | + | 0 |
| PGA3 | 11 | 60965984 | 60981850 | + | 47 |
| PGA4 | 11 | 60984821 | 61000679 | + | 66 |
| FADS1 | 11 | 61565597 | 61589529 | - | 6 |
| FTH1 | 11 | 61730257 | 61740132 | - | 6 |
| CBL | 11 | 119071986 | 119180359 | + | 0 |
| CD4 | 12 | 6893638 | 6931476 | + | 0 |
| CS | 12 | 56663983 | 56699175 | - | 6 |
| SHMT2 | 12 | 57618356 | 57630218 | + | 3 |
| METAP2 | 12 | 95862822 | 95911115 | + | 0 |
| MMAB | 12 | 109990020 | 110016358 | - | 0 |
| MVK | 12 | 110006500 | 110036571 | + | 7 |
| SDS | 12 | 113828750 | 113846692 | - | 31 |
| ATP12A | 13 | 25249549 | 25287423 | + | 0 |
| CLYBL | 13 | 100253919 | 100550888 | + | 0 |
| ATP4B | 13 | 114301619 | 114317513 | - | 0 |
| ABCD4 | 14 | 74750480 | 74774767 | - | 13 |
| AMN | 14 | 103383993 | 103398679 | + | 65 |
| TGM5 | 15 | 43523293 | 43564055 | - | 0 |
| DUT | 15 | 48618215 | 48637070 | + | 39 |
| NDUFAB1 | 16 | 23590835 | 23612639 | - | 22 |
| HP | 16 | 72083470 | 72096455 | + | 64 |
| HPR | 16 | 72092125 | 72112645 | + | 72 |
| PEMT | 17 | 17407377 | 17500434 | - | 0 |
| TNFAIP1 | 17 | 26657548 | 26675535 | + | 49 |
| GAST | 17 | 39863578 | 39873721 | + | 0 |
| ACLY | 17 | 40021669 | 40091795 | - | 95 |
| MMD | 17 | 53468474 | 53504341 | - | 0 |
| TYMS | 18 | 652590 | 674999 | + | 1 |
| MBP | 18 | 74689289 | 74849774 | - | 0 |
| FUT6 | 19 | 5829137 | 5844764 | - | 0 |
| CD320 | 19 | 8365511 | 8378439 | - | 0 |
| SLC27A1 | 19 | 17576300 | 17618477 | + | 0 |
| ATP4A | 19 | 36039595 | 36059560 | - | 0 |
| CD79A | 19 | 42376190 | 42386939 | + | 59 |
| FUT2 | 19 | 49194228 | 49210691 | + | 34 |
| FTL | 19 | 49462659 | 49471636 | + | 0 |
| NTSR1 | 20 | 61335189 | 61395623 | + | 0 |
| CBS | 21 | 44471801 | 44501472 | - | 2 |
| TCN2 | 22 | 30998070 | 31024547 | + | 0 |

^a^ Genes involved in B12 metabolism were from Pletscher-Frankild, Palleja, Tsafou, Binder, Jensen, 2015 ^36^ and Surendran et al., 2018 ^37^. The gene definitions included 5/1.5 kb relative to start/end coordinates in genes located in either the forward strand or reverse strands.

**Table S2.** Ninety genes related to vitamin B-12 levels and their functions (Szklarczyk et al., 2019) ^63^.

| Gene  (protein) | Function |
| --- | --- |
| TCN1  (transcobalamin-1) | Binds vitamin B12 with femtomolar affinity and protects it from the acidic environment of the stomach; Belongs to the eukaryotic cobalamin transport proteins family. (433 aa) |
| FUT6  (Alpha-(1,3)-fucosyltransferase 6) | Enzyme involved in the biosynthesis of the E-Selectin ligand, sialyl-Lewis X. Catalyzes the transfer of fucose from GDP- beta-fucose to alpha-2,3 sialylated substrates; Fucosyltransferases. (359 aa) |
| CUBN  (Cubilin/intrinsic-factor cobalamin receptor) | Cotransporter which plays a role in lipoprotein, vitamin and iron metabolism, by facilitating their uptake. Binds to ALB, MB, Kappa and lambda-light chains, TF, hemoglobin, GC, SCGB1A1, APOA1, high density lipoprotein, and the GIF-cobalamin complex. The binding of all ligands requires calcium. Serves as important transporter in several absorptive epithelia, including intestine, renal proximal tubules and embryonic yolk sac. Interaction with LRP2 mediates its trafficking throughout vesicles and facilitates the uptake of specific ligands like GC, hemoglobin, ALB, TF and SCGB1A1. (3623 aa) |
| LRP2  (Low density lipoprotein receptor-related protein 2) | Multiligand endocytic receptor (By similarity). Acts together with CUBN to mediate endocytosis of high-density lipoproteins (By similarity). Mediates receptor-mediated uptake of polybasic drugs such as aprotinin, aminoglycosides and polymyxin B (By similarity). In the kidney, mediates the tubular uptake and clearance of leptin (By similarity). Also mediates transport of leptin across the blood-brain barrier through endocytosis at the choroid plexus epithelium (By similarity). Endocytosis of leptin in neuronal cells is required for hypothalamic leptin signaling and leptin-mediated regulation of feeding and body weight (By similarity). Mediates endocytosis and subsequent lysosomal degradation of CST3 in kidney proximal tubule cells (By similarity). Mediates renal uptake of 25-hydroxyvitamin D3 in complex with the vitamin D3 transporter GC/DBP (By similarity). Mediates renal uptake of metallothionein-bound heavy metals (PubMed:15126248). Together with CUBN, mediates renal reabsorption of myoglobin (By similarity). Mediates renal uptake and subsequent lysosomal degradation of APOM (By similarity). Plays a role in kidney selenium homeostasis by mediating renal endocytosis of selenoprotein SEPP1 (By similarity). Mediates renal uptake of the antiapoptotic protein BIRC5/survivin which may be important for functional integrity of the kidney. Mediates renal uptake of matrix metalloproteinase MMP2 in complex with metalloproteinase inhibitor TIMP1 (By similarity). Mediates endocytosis of Sonic hedgehog protein N-product (ShhN), the active product of SHH (By similarity). Also mediates ShhN transcytosis (By similarity). In the embryonic neuroepithelium, mediates endocytic uptake and degradation of BMP4, is required for correct SHH localization in the ventral neural tube and plays a role in patterning of the ventral telencephalon (By similarity). Required at the onset of neurulation to sequester SHH on the apical surface of neuroepithelial cells of the rostral diencephalon ventral midline and to control PTCH1-dependent uptake and intracellular trafficking of SHH (By similarity). During neurulation, required in neuroepithelial cells for uptake of folate bound to the folate receptor FOLR1 which is necessary for neural tube closure (By similarity). In the adult brain, negatively regulates BMP signaling in the subependymal zone which enables neurogenesis to proceed (By similarity). In astrocytes, mediates endocytosis of ALB which is required for the synthesis of the neurotrophic factor oleic acid (By similarity). Involved in neurite branching (By similarity). During optic nerve development, required for SHH-mediated migration and proliferation of oligodendrocyte precursor cells (By similarity). Mediates endocytic uptake and clearance of SHH in the retinal margin which protects retinal progenitor cells from mitogenic stimuli and keeps them quiescent (By similarity). Plays a role in reproductive organ development by mediating uptake in reproductive tissues of androgen and estrogen bound to the sex hormone binding protein SHBG (By similarity). Mediates endocytosis of angiotensin-2 (By similarity). Also mediates endocytosis of angiotensis 1-7 (By similarity). Binds to the complex composed of beta-amyloid protein 40 and CLU/APOJ and mediates its endocytosis and lysosomal degradation (By similarity). Required for embryonic heart development (By similarity). Required for normal hearing, possibly through interaction with estrogen in the inner ear (By similarity). (4655 aa) |
| TCN2  (Transcobalamin 2) | Primary vitamin B12-binding and transport protein. Delivers cobalamin to cells; Belongs to the eukaryotic cobalamin transport proteins family. (427 aa) |
| PON1  (Serum paraoxonase/ arylesterase 1) | Hydrolyzes the toxic metabolites of a variety of organophosphorus insecticides. Capable of hydrolyzing a broad spectrum of organophosphate substrates and lactones, and a number of aromatic carboxylic acid esters. Mediates an enzymatic protection of low density lipoproteins against oxidative modification and the consequent series of events leading to atheroma formation; Belongs to the paraoxonase family. (355 aa) |
| SLC27A4  (Long-chain fatty acid transport protein 4) | Involved in translocation of long-chain fatty acids (LFCA) across the plasma membrane. Appears to be the principal fatty acid transporter in small intestinal enterocytes. Plays a role in the formation of the epidermal barrier. Required for fat absorption in early embryogenesis. Has acyl-CoA ligase activity for long-chain and very-long-chain fatty acids (VLCFAs). Indirectly inhibits RPE65 via substrate competition and via production of VLCFA derivatives like lignoceroyl-CoA. Prevents light-induced degeneration of rods and cones. (643 aa) |
| LXN  (Latexin) | Hardly reversible, non-competitive, and potent inhibitor of CPA1, CPA2 and CPA4. May play a role in inflammation; Belongs to the protease inhibitor I47 (latexin) family. (222 aa) |
| FPGS  (Folylpolyglutamate synthase) | Catalyzes conversion of folates to polyglutamate derivatives allowing concentration of folate compounds in the cell and the intracellular retention of these cofactors, which are important substrates for most of the folate-dependent enzymes that are involved in one-carbon transfer reactions involved in purine, pyrimidine and amino acid synthesis. Unsubstituted reduced folates are the preferred substrates. Metabolizes methotrexate (MTX) to polyglutamates; Belongs to the folylpolyglutamate synthase family. (587 aa) |
| PANK1  (Pantothenate kinase 1) | Plays a role in the physiological regulation of the intracellular CoA concentration; Belongs to the type II pantothenate kinase family. (598 aa) |
| PGA4  (Pepsin A-4) | Shows particularly broad specificity; although bonds involving phenylalanine and leucine are preferred, many others are also cleaved to some extent; Belongs to the peptidase A1 family. (388 aa) |
| MS4A3  (Membrane-spanning 4-domains subfamily A member 3) | Hematopoietic modulator for the G1-S cell cycle transition. Modulates the level of phosphorylation of cyclin- dependent kinase 2 (CDK2) through its direct binding to cyclin- dependent kinase inhibitor 3 (CDKN3/KAP); Belongs to the MS4A family. (214 aa) |
| TRDMT1  (tRNA (cytosine(38)-C(5))-methyltransferase) | Specifically methylates cytosine 38 in the anticodon loop of tRNA(Asp); Seven-beta-strand methyltransferase motif containing. (391 aa) |
| PGA3  (Pepsin A-3) | Shows particularly broad specificity; although bonds involving phenylalanine and leucine are preferred, many others are also cleaved to some extent. (388 aa) |
| FTH1  (Ferritin heavy chain) | Stores iron in a soluble, non-toxic, readily available form. Important for iron homeostasis. Has ferroxidase activity. Iron is taken up in the ferrous form and deposited as ferric hydroxides after oxidation. Also plays a role in delivery of iron to cells. Mediates iron uptake in capsule cells of the developing kidney (By similarity); Belongs to the ferritin family. (183 aa) |
| FTL  (Ferritin light chain) | Stores iron in a soluble, non-toxic, readily available form. Important for iron homeostasis. Iron is taken up in the ferrous form and deposited as ferric hydroxides after oxidation. Also plays a role in delivery of iron to cells. Mediates iron uptake in capsule cells of the developing kidney (By similarity); Belongs to the ferritin family. (175 aa) |
| FADS1  (Acyl-CoA (8-3)-desaturase) | Isoform 2 does not exhibit any catalytic activity toward 20:3n-6, but it may enhance FADS2 activity (By similarity). Isoform 1 is a component of a lipid metabolic pathway that catalyzes biosynthesis of highly unsaturated fatty acids (HUFA) from precursor essential polyunsaturated fatty acids (PUFA) linoleic acid (LA) (18:2n-6) and alpha-linolenic acid (ALA) (18:3n-3). Catalyzes the desaturation of dihomo-gamma-linoleic acid (DHGLA) (20:3n-6) and eicosatetraenoic acid (20:4n-3) to generate arachidonic acid (AA) (20:4n-6) and eicosapentaenoic acid (EPA)(20:5n-3), respectively. As a rate limiting enzyme for DGLA (20:3n-6) and AA (20:4n-6)-derived eicosanoid biosynthesis, controls the metabolism of inflammatory lipids like prostaglandin E2, critical for efficient acute inflammatory response and maintenance of epithelium homeostasis. Contributes to membrane phospholipid biosynthesis by providing AA (20:4n-6) as a major acyl chain esterified into phospholipids. In particular, regulates phosphatidylinositol-4,5-bisphosphate levels, modulating inflammatory cytokine production in T-cells (By similarity). Also desaturates (11E)-octadecenoate (trans-vaccenoate)(18:1n-9), a metabolite in the biohydrogenation pathway of LA (18:2n-6) (By similarity). (501 aa) |
| GIF  (Gastric intrinsic factor) | Promotes absorption of the essential vitamin cobalamin (Cbl) in the ileum. After interaction with CUBN, the GIF-cobalamin complex is internalized via receptor-mediated endocytosis. (417 aa) |
| MMADHC  (Methylmalonic aciduria and homocystinuria type D protein) | Involved in cobalamin metabolism. Plays a role in regulating the biosynthesis of two coenzymes, methylcobalamin and adenosylcobalamin. Plays a role in regulating the proportion of methylcobalamin and adenosylcobalamin. Promotes oxidation of cob(II)alamin bound to MMACHC. (296 aa) |
| CBL  (E3 ubiquitin-protein ligase) | Adapter protein that functions as a negative regulator of many signaling pathways that are triggered by activation of cell surface receptors. Acts as an E3 ubiquitin-protein ligase, which accepts ubiquitin from specific E2 ubiquitin-conjugating enzymes, and then transfers it to substrates promoting their degradation by the proteasome. Recognizes activated receptor tyrosine kinases, including KIT, FLT1, FGFR1, FGFR2, PDGFRA, PDGFRB, EGFR, CSF1R, EPHA8 and KDR and terminates signaling. Recognizes membrane-bound HCK, SRC and other kinases of the SRC family and mediates their ubiquitination and degradation. Participates in signal transduction in hematopoietic cells. Plays an important role in the regulation of osteoblast differentiation and apoptosis. Essential for osteoclastic bone resorption. The 'Tyr-731' phosphorylated form induces the activation and recruitment of phosphatidylinositol 3-kinase to the cell membrane in a signaling pathway that is critical for osteoclast function. May be functionally coupled with the E2 ubiquitin-protein ligase UB2D3. In association with CBLB, required for proper feedback inhibition of ciliary platelet-derived growth factor receptor-alpha (PDGFRA) signaling pathway via ubiquitination and internalization of PDGFRA (By similarity). (906 aa) |
| PNLIP  (Pancreatic triacylglycerol lipase) | Belongs to the AB hydrolase superfamily. Lipase family. (465 aa) |
| FUT2  (Galactoside alpha-(1,2)-fucosyltransferase 2) | Mediates the transfer of fucose to the terminal galactose on glycan chains of cell surface glycoproteins and glycolipids. The resulting epitope plays a role in cell-cell interaction including host-microbe interaction. Mediates interaction with intestinal microbiota influencing its composition. Creates a soluble precursor oligosaccharide FuC-alpha ((1,2)Galbeta-) called the H antigen which is an essential substrate for the final step in the soluble ABO blood group antigen synthesis pathway; Belongs to the glycosyltransferase 11 family. (343 aa) |
| CP  (Ceruloplasmin) | Ceruloplasmin is a blue, copper-binding (6-7 atoms per molecule) glycoprotein. It has ferroxidase activity oxidizing Fe(2+) to Fe(3+) without releasing radical oxygen species. It is involved in iron transport across the cell membrane. Provides Cu(2+) ions for the ascorbate-mediated deaminase degradation of the heparan sulfate chains of GPC1. May also play a role in fetal lung development or pulmonary antioxidant defense (By similarity). (1065 aa) |
| MMAA  (Methylmalonic aciduria type A protein) | GTPase, binds and hydrolyzes GTP. Involved in intracellular vitamin B12 metabolism, mediates the transport of cobalamin (Cbl) into mitochondria for the final steps of adenosylcobalamin (AdoCbl) synthesis. Functions as a G-protein chaperone that assists AdoCbl cofactor delivery from MMAB to the methylmalonyl-CoA mutase (MUT) and reactivation of the enzyme during catalysis. (418 aa) |
| MMACHC  (Methylmalonic aciduria and homocystinuria type C protein) | Catalyzes the reductive dealkylation of cyanocobalamin to cob(II)alamin, using FAD or FMN as cofactor and NADPH as cosubstrate. Can also catalyze the glutathione-dependent reductive demethylation of methylcobalamin, and, with much lower efficiency, the glutathione-dependent reductive demethylation of adenosylcobalamin. Under anaerobic conditions cob(I)alamin is the first product; it is highly reactive and is converted to aquocob(II)alamin in the presence of oxygen. Binds cyanocobalamin, adenosylcobalamin, methylcobalamin and others. (282 aa) |
| LMBRD1  (Lysosomal cobalamin transport escort protein LMBD1) | Probable lysosomal cobalamin transporter. Required to export cobalamin from lysosomes allowing its conversion to cofactors. Isoform 3 may play a role in the assembly of hepatitis delta virus (HDV). (540 aa) |
| TFRC  (Transferrin receptor protein 1) | Cellular uptake of iron occurs via receptor-mediated endocytosis of ligand-occupied transferrin receptor into specialized endosomes. Endosomal acidification leads to iron release. The apotransferrin-receptor complex is then recycled to the cell surface with a return to neutral pH and the concomitant loss of affinity of apotransferrin for its receptor. Transferrin receptor is necessary for development of erythrocytes and the nervous system (By similarity). A second ligand, the heditary hemochromatosis protein HFE, competes for binding with transferrin for an overlapping C-terminal binding site. Positively regulates T and B cell proliferation through iron uptake. Acts as a lipid sensor that regulates mitochondrial fusion by regulating activation of the JNK pathway. When dietary levels of stearate (C18:0) are low, promotes activation of the JNK pathway, resulting in HUWE1-mediated ubiquitination and subsequent degradation of the mitofusin MFN2 and inhibition of mitochondrial fusion. When dietary levels of stearate (C18:0) are high, TFRC stearoylation inhibits activation of the JNK pathway and thus degradation of the mitofusin MFN2. (760 aa) |
| SARS  (Serine--tRNA ligase) | Catalyzes the attachment of serine to tRNA(Ser) in a two-step reaction: serine is first activated by ATP to form Ser- AMP and then transferred to the acceptor end of tRNA(Ser). Is probably also able to aminoacylate tRNA(Sec) with serine, to form the misacylated tRNA L-seryl-tRNA(Sec), which will be further converted into selenocysteinyl-tRNA(Sec). In the nucleus, binds to the VEGFA core promoter and prevents MYC binding and transcriptional activation by MYC. Recruits SIRT2 to the VEGFA promoter, promoting deacetylation of histone H4 at 'Lys-16' (H4K16). Thereby, inhibits the production of VEGFA and sprouting angiogenesis mediated by VEGFA. (514 aa) |
| MUT  (methylmalonyl CoA mutase) | Involved in the degradation of several amino acids, odd- chain fatty acids and cholesterol via propionyl-CoA to the tricarboxylic acid cycle. MCM has different functions in other species; Belongs to the methylmalonyl-CoA mutase family. (750 aa) |
| GNMT  (Glycine N-methyltransferase) | Catalyzes the methylation of glycine by using S- adenosylmethionine (AdoMet) to form N-methylglycine (sarcosine) with the concomitant production of S-adenosylhomocysteine (AdoHcy). Possible crucial role in the regulation of tissue concentration of AdoMet and of metabolism of methionine; Seven-beta-strand methyltransferase motif containing. (295 aa) |
| HAT1  (Histone acetyltransferase type B catalytic subunit) | Acetylates soluble but not nucleosomal histone H4 at 'Lys-5' (H4K5ac) and 'Lys-12' (H4K12ac) and, to a lesser extent, acetylates histone H2A at 'Lys-5' (H2AK5ac). Has intrinsic substrate specificity that modifies lysine in recognition sequence GXGKXG. May be involved in nucleosome assembly during DNA replication and repair as part of the histone H3.1 and H3.3 complexes. May play a role in DNA repair in response to free radical damage; Belongs to the HAT1 family. (419 aa) |
| MTHFR  (5-methyl-tetrahydrafolate reductase) | Catalyzes the conversion of 5,10- methylenetetrahydrofolate to 5-methyltetrahydrofolate, a co- substrate for homocysteine remethylation to methionine. (656 aa) |
| DUT  (Deoxyuridine 5'-triphosphate nucleotidohydrolase) | This enzyme is involved in nucleotide metabolism: it produces dUMP, the immediate precursor of thymidine nucleotides and it decreases the intracellular concentration of dUTP so that uracil cannot be incorporated into DNA; Belongs to the dUTPase family. (252 aa) |
| CASR  (Extracellular calcium-sensing receptor) | G-protein-coupled receptor that senses changes in the extracellular concentration of calcium ions and plays a key role in maintaining calcium homeostasis. Senses fluctuations in the circulating calcium concentration and modulates the production of parathyroid hormone (PTH) in parathyroid glands (By similarity). The activity of this receptor is mediated by a G-protein that activates a phosphatidylinositol- calcium second messenger system. The G-protein- coupled receptor activity is activated by a co-agonist mechanism: aromatic amino acids, such as Trp or Phe, act concertedly with divalent cations, such as calcium or magnesium, to achieve full receptor activation. (1088 aa) |
| METAP2  (Methionine aminopeptidase 2) | Cotranslationally removes the N-terminal methionine from nascent proteins. The N-terminal methionine is often cleaved when the second residue in the primary sequence is small and uncharged (Met-Ala-, Cys, Gly, Pro, Ser, Thr, or Val). The catalytic activity of human METAP2 toward Met-Val peptides is consistently two orders of magnitude higher than that of METAP1, suggesting that it is responsible for processing proteins containing N- terminal Met-Val and Met-Thr sequences in vivo; Belongs to the peptidase M24A family. Protects eukaryotic initiation factor EIF2S1 from translation-inhibiting phosphorylation by inhibitory kinases such as EIF2AK2/PKR and EIF2AK1/HCR. Plays a critical role in the regulation of protein synthesis. (478 aa) |
| PGC  (Gastricsin) | Gastricsin; Hydrolyzes a variety of proteins; Belongs to the peptidase A1 family. (388 aa) |
| METAP1D  (Methionine aminopeptidase 1D) | Removes the N-terminal methionine from nascent proteins. The N-terminal methionine is often cleaved when the second residue in the primary sequence is small and uncharged (Met-Ala-, Cys, Gly, Pro, Ser, Thr, or Val). Requires deformylation of the N(alpha)-formylated initiator methionine before it can be hydrolyzed (By similarity). May play a role in colon tumorigenesis; Belongs to the peptidase M24A family. Methionine aminopeptidase type 1 subfamily. (335 aa) |
| UBIAD1  (UbiA prenyltransferase domain-containing protein 1) | Prenyltransferase that mediates the formation of menaqui--4 (MK-4) and coenzyme Q10. MK-4 is a vitamin K2 isoform present at high concentrations in the brain, kidney and pancreas, and is required for endothelial cell development. Mediates the conversion of phylloqui- (PK) into MK-4, probably by cleaving the side chain of phylloqui- (PK) to release 2- methyl-1,4-naphthoqui- (menadione; K3) and then prenylating it with geranylgeranyl pyrophosphate (GGPP) to form MK-4. Also plays a role in cardiovascular development independently of MK-4 biosynthesis, by acting as a coenzyme Q10 biosynthetic enzyme: coenzyme Q10, also named ubiqui-, plays an important antioxidant role in the cardiovascular system. Mediates biosynthesis of coenzyme Q10 in the Golgi membrane, leading to protect cardiovascular tissues from NOS3/eNOS-dependent oxidative stress. (338 aa) |
| ACOT7  (Cytosolic acyl coenzyme A thioester hydrolase) | Acyl-CoA thioesterases are a group of enzymes that catalyze the hydrolysis of acyl-CoAs to the free fatty acid and coenzyme A (CoASH), providing the potential to regulate intracellular levels of acyl-CoAs, free fatty acids and CoASH. May play an important physiological function in brain. May play a regulatory role by modulating the cellular levels of fatty acyl- CoA ligands for certain transcription factors as well as the substrates for fatty acid metabolizing enzymes, contributing to lipid homeostasis. (380 aa) |
| ATP4B  (Potassium-transporting ATPase subunit beta) | Required for stabilization and maturation of the catalytic proton pump alpha subunit and may also involved in cell adhesion and establishing epithelial cell polarity; ATPase H+/K+ transporting. (291 aa) |
| GYPA  (Glycophorin-A) | Glycophorin A is the major intrinsic membrane protein of the erythrocyte. The N-terminal glycosylated segment, which lies outside the erythrocyte membrane, has MN blood group receptors. Appears to be important for the function of SLC4A1 and is required for high activity of SLC4A1. May be involved in translocation of SLC4A1 to the plasma membrane. Is a receptor for influenza virus. Is a receptor for Plasmodium falciparum erythrocyte-binding antigen 175 (EBA-175); binding of EBA-175 is dependent on sialic acid residues of the O-linked glycans. Appears to be a receptor for Hepatitis A virus (HAV). (150 aa) |
| TGM5  (Protein-glutamine gamma-glutamyltransferase 5) | Catalyzes the cross-linking of proteins and the conjugation of polyamines to proteins. Contributes to the formation of the cornified cell envelope of keratinocytes; Belongs to the transglutaminase superfamily. Transglutaminase Family. (720 aa) |
| HES2  (Transcription factor HES-2) | Transcriptional repressor of genes that require a bHLH protein for their transcription; Basic helix-loop-helix proteins. (173 aa) |
| ACLY  (ATP-citrate synthase) | ATP-citrate synthase is the primary enzyme responsible for the synthesis of cytosolic acetyl-CoA in many tissues. Has a central role in de novo lipid synthesis. In nervous tissue it may be involved in the biosynthesis of acetylcholine; In the N-terminal section; belongs to the succinate/malate CoA ligase beta subunit family. (1101 aa) |
| GAST  (Gastrin) | Gastrin stimulates the stomach mucosa to produce and secrete hydrochloric acid and the pancreas to secrete its digestive enzymes. It also stimulates smooth muscle contraction and increases blood circulation and water secretion in the stomach and intestine; Endogenous ligands. (101 aa) |
| TF  (Serotransferrin) | Transferrins are iron binding transport proteins which can bind two Fe(3+) ions in association with the binding of an anion, usually bicarbonate. It is responsible for the transport of iron from sites of absorption and heme degradation to those of storage and utilization. Serum transferrin may also have a further role in stimulating cell proliferation. (698 aa) |
| CD79A  (B-cell antigen receptor complex-associated protein alpha chain) | Required in cooperation with CD79B for initiation of the signal transduction cascade activated by binding of antigen to the B-cell antigen receptor complex (BCR) which leads to internalization of the complex, trafficking to late endosomes and antigen presentation. Also required for BCR surface expression and for efficient differentiation of pro- and pre-B-cells. Stimulates SYK autophosphorylation and activation. Binds to BLNK, bringing BLNK into proximity with SYK and allowing SYK to phosphorylate BLNK. Also interacts with and increases activity of some Src-family tyrosine kinases. Represses BCR signaling during development of immature B-cells. (226 aa) |
| ADAMTS2  (A disintegrin and metalloproteinase with thrombospondin motifs 2) | Cleaves the propeptides of type I and II collagen prior to fibril assembly. Does not act on type III collagen. May also play a role in development that is independent of its role in collagen biosynthesis; ADAM metallopeptidases with thrombospondin type 1 motif. (1211 aa) |
| ATP12A  (Potassium-transporting ATPase alpha chain 2) | Catalyzes the hydrolysis of ATP coupled with the exchange of H(+) and K(+) ions across the plasma membrane. Responsible for potassium absorption in various tissues; Belongs to the cation transport ATPase (P-type) (TC 3.A.3) family. Type IIC subfamily. (1045 aa) |
| NTSR1  (Neurotensin receptor type 1) | G-protein coupled receptor for the tridecapeptide neurotensin (NTS). Signaling is effected via G proteins that activate a phosphatidylinositol-calcium second messenger system. Signaling leads to the activation of downstream MAP kinases and protects cells against apoptosis. (418 aa) |
| MBP  (Myelin basic protein) | The classic group of MBP isoforms (isoform 4-isoform 14) are with PLP the most abundant protein components of the myelin membrane in the CNS. They have a role in both its formation and stabilization. The smaller isoforms might have an important role in remyelination of denuded axons in multiple sclerosis. The non- classic group of MBP isoforms (isoform 1-isoform 3/Golli-MBPs) may preferentially have a role in the early developing brain long before myelination, maybe as components of transcriptional complexes, and may also be involved in signaling pathways in T-cells and neural cells. Differential splicing events combined with optional post-translational modifications give a wide spectrum of isomers, with each of them potentially having a specialized function. Induces T-cell proliferation. (197 aa) |
| IFITM2  (Interferon-induced transmembrane protein 2) | IFN-induced antiviral protein which inhibits the entry of viruses to the host cell cytoplasm, permitting endocytosis, but preventing subsequent viral fusion and release of viral contents into the cytosol. Active against multiple viruses, including influenza A virus, SARS coronavirus (SARS-CoV), Marburg virus (MARV), Ebola virus (EBOV), Dengue virus (DNV), West Nile virus (WNV), human immunodeficiency virus type 1 (HIV-1) and vesicular stomatitis virus (VSV). Can inhibit: influenza virus hemagglutinin protein-mediated viral entry, MARV and EBOV GP1,2-mediated viral entry, SARS-CoV S protein-mediated viral entry and VSV G protein-mediated viral entry. Induces cell cycle arrest and mediates apoptosis by caspase activation and in p53-independent manner. (132 aa) |
| ABCD4  (ATP Binding Cassette Subfamily D Member) | May be involved in intracellular processing of vitamin B12 (cobalamin). Could play a role in the lysosomal release of vitamin B12 into the cytoplasm; ATP binding cassette subfamily D. (606 aa) |
| TNFAIP1  (BTB/ POZ domain-containing adapter for CUL3-mediated RhoA degradation protein 2) | Substrate-specific adapter of a BCR (BTB-CUL3-RBX1) E3 ubiquitin-protein ligase complex involved in regulation of cytoskeleton structure. The BCR(TNFAIP1) E3 ubiquitin ligase complex mediates the ubiquitination of RHOA, leading to its degradation by the proteasome, thereby regulating the actin cytoskeleton and cell migration. Its interaction with RHOB may regulate apoptosis. May enhance the PCNA-dependent DNA polymerase delta activity; Belongs to the BACURD family. (316 aa) |
| PEMT  (Phosphatidylethanolamine N-methyltransferase) | Catalyzes the three sequential steps of the methylation pathway of phosphatidylcholine biosynthesis, the SAM-dependent methylation of phosphatidylethanolamine (PE) to phosphatidylmonomethylethanolamine (PMME), PMME to phosphatidyldimethylethanolamine (PDME), and PDME to phosphatidylcholine (PC); Belongs to the class VI-like SAM-binding methyltransferase superfamily. PEMT/PEM2 methyltransferase family. (236 aa) |
| CLYBL  (Citrate lyase beta like) | Mitochondrial citramalyl-CoA lyase indirectly involved in the vitamin B12 metabolism. Converts citramalyl-CoA into acetyl-CoA and pyruvate in the C5- dicarboxylate catabolism pathway. The C5- dicarboxylate catabolism pathway is required to detoxify itaconate, a vitamin B12-poisoning metabolite. Also acts as a malate synthase in vitro, converting glyoxylate and acetyl-CoA to malate. Also acts as a beta-methylmalate synthase in vitro, by mediating conversion of glyoxylate and propionyl-CoA to beta-methylmalate. Also has very weak citramalate synthase activity in vitro. (340 aa) |
| NDUFAB1  (Acyl carrier protein) | Carrier of the growing fatty acid chain in fatty acid biosynthesis (By similarity). Accessory and non-catalytic subunit of the mitochondrial membrane respiratory chain NADH dehydrogenase (Complex I), which functions in the transfer of electrons from NADH to the respiratory chain; Belongs to the acyl carrier protein (ACP) family. (156 aa) |
| MTR  (5-methyltetrahydrofolate-homocysteine methyltransferase) | Catalyzes the transfer of a methyl group from methyl- cobalamin to homocysteine, yielding enzyme-bound cob(I)alamin and methionine. Subsequently, remethylates the cofactor using methyltetrahydrofolate (By similarity); Belongs to the vitamin-B12 dependent methionine synthase family. (1265 aa) |
| METAP1  (Methionine aminopeptidase 1) | Cotranslationally removes the N-terminal methionine from nascent proteins. The N-terminal methionine is often cleaved when the second residue in the primary sequence is small and uncharged (Met-Ala-, Cys, Gly, Pro, Ser, Thr, or Val). Required for normal progression through the cell cycle; Belongs to the peptidase M24A family. Methionine aminopeptidase type 1 subfamily. (386 aa) |
| CRP  (C-reactive protein) | Displays several functions associated with host defense: it promotes agglutination, bacterial capsular swelling, phagocytosis and complement fixation through its calcium-dependent binding to phosphorylcholine. Can interact with DNA and histones and may scavenge nuclear material released from damaged circulating cells; Short pentraxins. (224 aa) |
| CBS  (Cystathionine beta synthase) | Hydro-lyase catalyzing the first step of the transsulfuration pathway, where the hydroxyl group of L-serine is displaced by L-homocysteine in a beta-replacement reaction to form L-cystathionine, the precursor of L-cysteine. This catabolic route allows the elimination of L-methionine and the toxic metabolite L-homocysteine. Also involved in the production of hydrogen sulfide, a gasotransmitter with signaling and cytoprotective effects on neurons. (551 aa) |
| CD4  (T-cell surface glycoprotein CD4) | Integral membrane glycoprotein that plays an essential role in the immune response and serves multiple functions in responses against both external and internal offenses. In T-cells, functions primarily as a coreceptor for MHC class II molecule:peptide complex. The antigens presented by class II peptides are derived from extracellular proteins while class I peptides are derived from cytosolic proteins. Interacts simultaneously with the T-cell receptor (TCR) and the MHC class II presented by antigen presenting cells (APCs). In turn, recruits the Src kinase LCK to the vicinity of the TCR-CD3 complex. LCK then initiates different intracellular signaling pathways by phosphorylating various substrates ultimately leading to lymphokine production, motility, adhesion and activation of T-helper cells. In other cells such as macrophages or NK cells, plays a role in differentiation/activation, cytokine expression and cell migration in a TCR/LCK-independent pathway. Participates in the development of T-helper cells in the thymus and triggers the differentiation of monocytes into functional mature macrophages. (458 aa) |
| CSN1S1  (Alpha-S1-casein) | Important role in the capacity of milk to transport calcium phosphate; Belongs to the alpha-casein family. (185 aa) |
| MVK  (Mevalonate kinase) | May be a regulatory site in cholesterol biosynthetic pathway; Belongs to the GHMP kinase family. Mevalonate kinase subfamily. (396 aa) |
| SCP2  (Non-specific lipid-transfer protein) | Mediates in vitro the transfer of all common phospholipids, cholesterol and gangliosides between membranes. May play a role in regulating steroidogenesis. (547 aa) |
| CTH  (Cystathionine gamma-lyase) | Catalyzes the last step in the trans-sulfuration pathway from methionine to cysteine. Has broad substrate specificity. Converts cystathionine to cysteine, ammonia and 2-oxobutanoate. Converts two cysteine molecules to lanthionine and hydrogen sulfide. Can also accept homocysteine as substrate. Specificity depends on the levels of the endogenous substrates. Generates the endogenous signaling molecule hydrogen sulfide (H2S), and so contributes to the regulation of blood pressure. Acts as a cysteine-protein sulfhydrase by mediating sulfhydration of target proteins: sulfhydration consists of converting -SH groups into -SSH on specific cysteine residues of target proteins such as GAPDH, PTPN1 and NF-kappa-B subunit RELA, thereby regulating their function. (405 aa) |
| F3  (Tissue fator) | Initiates blood coagulation by forming a complex with circulating factor VII or VIIa. The [TF:VIIa] complex activates factors IX or X by specific limited protolysis. TF plays a role in normal hemostasis by initiating the cell-surface assembly and propagation of the coagulation protease cascade. (295 aa) |
| LIN9  (Protein lin-9 homolog) | Acts as a tumor suppressor. Inhibits DNA synthesis. Its ability to inhibit oncogenic transformation is mediated through its association with RB1. Plays a role in the expression of genes required for the G1/S transition; Belongs to the lin-9 family. (558 aa) |
| THUMPD2  (THUMP domain-containing protein 2) | Seven-beta-strand methyltransferase motif containing; Belongs to the methyltransferase superfamily. (503 aa) |
| TXNDC9  (Thioredoxin domain-containing protein 9) | Significantly diminishes the chaperonin TCP1 complex ATPase activity, thus negatively impacts protein folding, including that of actin or tubulin. (226 aa) |
| ALDH1L1  (Cytosolic 10-formyltetrahydrofolate dehydrogenase) | Aldehyde dehydrogenase 1 family member L1; In the N-terminal section; belongs to the GART family. (912 aa) |
| HTN3  (Histatin-3) | Histatins are salivary proteins that are considered to be major precursors of the protective proteinaceous structure on tooth surfaces (enamel pellicle). In addition, histatins exhibit antibacterial and antifungal activities. His3-(20-43)-peptide (histatin-5) is especially effective against C.albicans and C.neoformans, and inhibits Lys-gingipain and Arg-gingipain (rgpB) from P.gingivalis. In addition, His3-(20-43)-peptide is a potent inhibitor of metalloproteinases MMP2 and MMP9. (51 aa) |
| ALB  (Albumin) | Serum albumin, the main protein of plasma, has a good binding capacity for water, Ca(2+), Na(+), K(+), fatty acids, hormones, bilirubin and drugs. Its main function is the regulation of the colloidal osmotic pressure of blood. Major zinc transporter in plasma, typically binds about 80% of all plasma zinc; Belongs to the ALB/AFP/VDB family. (609 aa) |
| MTRR  (Methionine synthase reductase) | Involved in the reductive regeneration of cob(I)alamin (vitamin B12) cofactor required for the maintenance of methionine synthase in a functional state. Necessary for utilization of methylgroups from the folate cycle, thereby affecting transgenerational epigenetic inheritance. Folate pathway donates methyl groups necessary for cellular methylation and affects different pathways such as DNA methylation, possibly explaining the transgenerational epigenetic inheritance effects. (725 aa) |
| PRELID2  (PRELI domain-containing protein 2) | Phosphatidic acid transfer activity. Phospholipid transport. (189 aa) |
| HRH2  (Histamine H2 receptor) | The H2 subclass of histamine receptors mediates gastric acid secretion. Also appears to regulate gastrointestinal motility and intestinal secretion. Possible role in regulating cell growth and differentiation. The activity of this receptor is mediated by G proteins which activate adenylyl cyclase and, through a separate G protein-dependent mechanism, the phosphoinositide/protein kinase (PKC) signaling pathway (By similarity); Belongs to the G-protein coupled receptor 1 family. (397 aa) |
| GGH  (Gamma-glutamyl hydrolase) | Hydrolyzes the polyglutamate sidechains of pteroylpolyglutamates. Progressively removes gamma-glutamyl residues from pteroylpoly-gamma-glutamate to yield pteroyl-alpha- glutamate (folic acid) and free glutamate. May play an important role in the bioavailability of dietary pteroylpolyglutamates and in the metabolism of pteroylpolyglutamates and antifolates; Belongs to the peptidase C26 family. (318 aa) |
| CS  (Citrate synthase) | Catalytic activity. This protein is involved in step 1 of the subpathway that synthesizes isocitrate from oxaloacetate. Citrate synthase is found in nearly all cells capable of oxidative metabolism. (466 aa) |
| SHMT2  (Serine hydroxymethyltransferase) | Contributes to the de novo mitochondrial thymidylate biosynthesis pathway via its role in glycine and tetrahydrofolate metabolism. Thymidylate biosynthesis is required to prevent uracil accumulation in mtDNA. Interconversion of serine and glycine. Associates with mitochondrial DNA. Plays a role in the deubiquitination of target proteins as component of the BRISC complex. Required for IFNAR1 deubiquitination by the BRISC complex; Belongs to the SHMT family. (504 aa) |
| MMAB  (Corrinoid adenosyltransferase) | Adenosyltransferase involved in intracellular vitamin B12 metabolism. Generates adenosylcobalamin (AdoCbl) and directly delivers the cofactor to MUT in a transfer taht is stimulated by ATP-binding to MMAB and gated by MMAA; Cilia and flagella associated. (250 aa) |
| SDS  (L-serine dehydratase/L-threonine deaminase) | Catalytic activity. Serine dehydratase. (328 aa) |
| AMN  (Protein amnionless) | Necessary for efficient absorption of vitamin B12. Required for normal CUBN- mediated protein transport in the kidney. May direct the production of trunk mesoderm during development by modulating a bone morphogenetic protein (BMP) signaling pathway in the underlying visceral endoderm (By similarity). (453 aa) |
| HP  (Haptoglobin) | As a result of hemolysis, hemoglobin is found to accumulate in the kidney and is secreted in the urine. Haptoglobin captures, and combines with free plasma hemoglobin to allow hepatic recycling of heme iron and to prevent kidney damage. Haptoglobin also acts as an Antimicrobial; Antioxidant, has antibacterial activity and plays a role in modulating many aspects of the acute phase response. Hemoglobin/haptoglobin complexes are rapidely cleared by the macrophage CD163 scavenger receptor expressed on the surface of liver Kupfer cells through an endocytic lysosomal degradation pathway. (406 aa) |
| HPR  (Haptoglobin-related protein) | Primate-specific plasma protein associated with apolipoprotein L-I (apoL-I)-containing high-density lipoprotein (HDL). This HDL particle, termed trypanosome lytic factor-1 (TLF- 1), mediates human innate immune protection against many species of African trypanosomes. Binds hemoglobin with high affinity and may contribute to the clearance of cell-free hemoglobin to allow hepatic recycling of heme iron; Sushi domain containing. (348 aa) |
| MMD  (Monocyte to macrophage differentiation factor) | Involved in the dynamics of lysosomal membranes associated with microglial activation following brain lesion; Progestin and adipoQ receptor family. (238 aa) |
| TYMS  (Thymidylate synthase) | Contributes to the de novo mitochondrial thymidylate biosynthesis pathway. (313 aa) |
| CD320  (CD320 molecule) | Receptor for transcobalamin saturated with cobalamin (TCbl). Plays an important role in cobalamin uptake. Plasma membrane protein that is expressed on follicular dendritic cells (FDC) and mediates interaction with germinal center B cells. Functions as costimulator to promote B cell responses to antigenic stimuli; promotes B cell differentiation and proliferation. Germinal center-B (GC-B) cells differentiate into memory B-cells and plasma cells (PC) through interaction with T-cells and follicular dendritic cells (FDC). CD320 augments the proliferation of PC precursors generated by IL-10. (282 aa) |
| SLC27A1  (Long-chain fatty acid transport protein 1) | Involved in translocation of long-chain fatty acids (LFCA) across the plasma membrane. The LFCA import appears to be hormone-regulated in a tissue-specific manner. In adipocytes, but not myocytes, insulin induces a rapid translocation of FATP1 from intracellular compartments to the plasma membrane, paralleled by increased LFCA uptake. May act directly as a bona fide transporter, or alternatively, in a cytoplasmic or membrane- associated multimeric protein complex to trap and draw fatty acids towards accumulation. Plays a pivotal role in regulating available LCFA substrates from exogenous sources in tissues undergoing high levels of beta-oxidation or triglyceride synthesis. May be involved in regulation of cholesterol metabolism (By similarity). Probably involved in fatty acid transport across the blood barrier. (646 aa) |
| ATP4A  (Potassium-transporting ATPase alpha chain 1) | Catalyzes the hydrolysis of ATP coupled with the exchange of H(+) and K(+) ions across the plasma membrane. Responsible for acid production in the stomach; ATPase H+/K+ transporting. (1035 aa) |

**Table S3.** SNP_ref_ associated with B-12 levels after correction for multiple testing and their SNP_Block_^a^.

| SNP_ref_ | Gene Symbol | SNP_Block_ |
| --- | --- | --- |
| brz:22:31023366:AG | TCN2 | - |
| rs1304811491 | ADAMTS2 | rs933975928, brz:5:178772439:AC |
| rs147671744 | PEMT | - |
| rs72648013 | MVK | - |
| rs12081406 | SARS | rs55712540 |
| rs2182598 | SARS | rs72973290, rs61751058, rs77400401 |
| rs10489132 | LXN | - |
| rs116157801 | FPGS | rs116157801, rs73614240, rs115146331 |
| rs28989521 | CD4 | rs11064396 |
| rs144213212 | SLC27A4 | - |
| rs721922 | CLYBL | rs1924099, rs7332098, rs34667146, rs12232034, rs9557316, rs56197435, rs72653983, rs17612087, rs72653992 |
| rs74791051 | LRP2 | rs41268685, rs41268685, rs147335778 |
| rs41288280 | ATP12A | - |
| rs10489132 | METAP1 | rs11097642 |
| rs116910911 | MBP | rs470695,rs72990916,rs77870478 |
| rs75243301 | FUT6 | rs61740561, rs61739552, rs141349215, rs73536616, rs73536629 |
| rs117412228 | CUBN | - |
| rs10925263 | MTR | rs28372869, rs28372870, rs3738547, rs3738547, rs73119852, rs10495385, rs77226497, rs80326214 ,rs113277607 ,rs113277607, rs7526063, rs6682427, rs79359872, rs7541539, rs75328033, rs77741950, rs10925251, rs73129187, rs3820569, rs541441537, rs10495387, rs3768154, rs77775662 ,rs12049580, rs12562226, rs16834527, rs12022937, rs73131123, rs73131123, rs3768159, rs3768160, rs3768160, rs3768161, rs61400757, rs59514993, rs6677090, rs6679990, rs6679990, rs16834541, rs14354, rs14354, rs3738551 |
| rs111386779 | CBS | rs73372393, rs73372396 |
| rs11254386 | CUBN | rs73600122, rs73600202, rs73602209, rs12265457, rs116231647, rs146910247, rs146910247, rs10159806, rs10159806, rs139580342 |
| rs116286548 | TCN1 | - |
| rs1220841 | CSN1S1 | rs776851 |
| rs138583897 | LRP2 | - |
| rs141206548 | LMBRD1 | rs112053239 |
| rs144147038 | LRP2 | - |
| rs148126159 | ABCD4 | - |
| rs150181241 | CBS | - |
| rs1529038 | CSN1S1 | rs17696835, rs962689, rs62305467, rs62305467, rs1231466, rs1237375, rs1002081, rs776847, rs17699715, rs13123938, rs776852, rs6600804 |
| rs1800249 | TF | rs8177191, rs8177217, rs8177221, rs3811657 |
| rs2808631 | CRP | - |
| rs34864360 | NTSR1 | rs78644145, rs16983222, rs16983222, rs144839366, rs143151412, rs143151412, rs146672963, rs146672963, rs141474602 |
| rs45613333 | NTSR1 | - |
| rs6090388 | NTSR1 | rs6122428, rs6090389 |
| rs7633232 | TF | rs6785596 |
| rs79752143 | NDUFAB1 | - |
| rs8083543 | MBP | - |

^a^ SNP_Block_ (r^2^ > 0.2) with 36 SNP_ref._
